# Characterizing the Movement of Per- and Polyfluoroalkyl Substances in an Avian Aquatic–Terrestrial Food Web

**DOI:** 10.1021/acs.est.3c06944

**Published:** 2023-11-24

**Authors:** Kailee E. Hopkins, Melissa A. McKinney, Amandeep Saini, Robert J. Letcher, Natalie K. Karouna-Renier, Kim J. Fernie

**Affiliations:** † Ecotoxicology and Wildlife Health Division, Environment and Climate Change Canada, 867 Lakeshore Road, Burlington, ON L7R 4A6, Canada; ‡ Department of Natural Resource Sciences, McGill University, 21111 Lakeshore Road, Sainte-Anne-de-Bellevue, QC H9X 3V9, Canada; § Air Quality Processes Research Section, Environment and Climate Change Canada, 4905 Dufferin Street, North York, ON M3H 5T4, Canada; ∥ Ecotoxicology and Wildlife Health Division, Environment and Climate Change Canada, Carleton University, 1125 Colonel By Drive, Ottawa, ON K1A 0H3, Canada; ⊥ U.S. Geological Survey, Eastern Ecological Science Center, Patuxent Research Refuge, 12302 Beech Forest Road, Laurel, Maryland 20708, United States

**Keywords:** tree swallows, perfluoroalkyl acids, fatty acid signatures, bioaccumulation, biomagnification, invertebrates, environmental sources

## Abstract

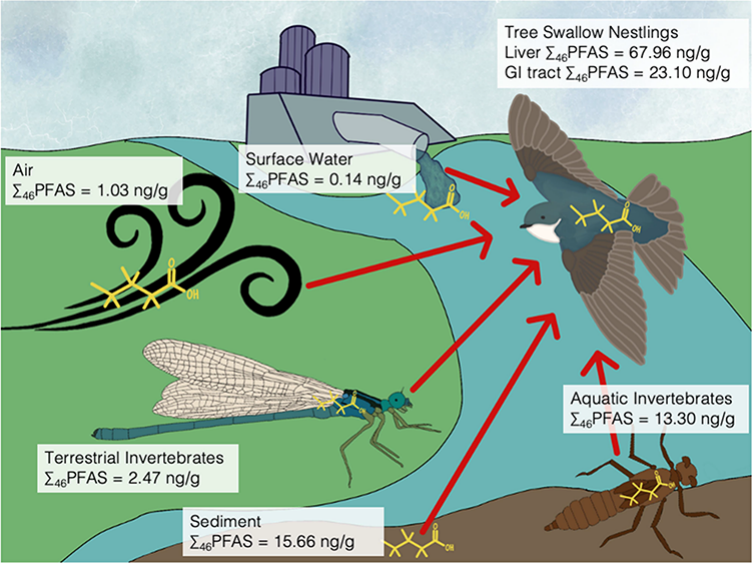

The movement of per- and polyfluoroalkyl substances (PFAS) through linked aquatic–terrestrial food webs is not well understood. Tree swallows (*Tachycineta bicolor*) in such systems may be exposed to PFAS from multiple abiotic and/or biotic compartments. We show from fatty acid signatures and carbon stable isotopes that tree swallow nestlings in southwestern Ontario fed on both terrestrial and aquatic macroinvertebrates. The PFAS profiles of air, terrestrial invertebrates, and swallows were dominated by perfluorooctanesulfonic acid (PFOS). Short-chain perfluoroalkyl acids (PFAAs) were largely restricted to air, surface water, and sediment, and long-chain PFAAs were mainly found in aquatic invertebrates and tree swallows. PFOS, multiple long-chain perfluorocarboxylic acids [perfluorononanoic acid (PFNA), perfluorodecanoic acid (PFDA), perfluorotridecanoic acid (PFTrDA)] and perfluorooctane sulfonamide precursors were estimated to bioaccumulate from air to tree swallows. PFOS bioaccumulated from air to terrestrial invertebrates, and PFOS, PFDA, and perfluorooctane sulfonamidoacetic acids (FOSAAs) bioaccumulated from water to aquatic invertebrates. PFOS showed biomagnification from both terrestrial and aquatic invertebrates to tree swallows, and PFDA and FOSAAs were also biomagnified from aquatic invertebrates to tree swallows. The movement of PFAS through aquatic–terrestrial food webs appears congener- and compartment-specific, challenging the understanding of PFAS exposure routes for multiple species involved in these food webs.

## Abstract 2

Perfluoroalkyl acid congeners and some precursors bioaccumulate and biomagnify to nestling tree swallows from dietary consumption of aquatic invertebrates and possibly inhalation thereby characterizing PFAS movements through aquatic−terrestrial food webs.

## Introduction

Per- and polyfluoroalkyl substances (PFAS) are synthetic organic chemicals found ubiquitously in the environment because of their high chemical and thermal stability,^1,2^ and are widely used in industrial applications and consumer products.^3^ PFAS include the perfluoroalkyl acids (PFAAs) [e.g., the perfluoroalkyl carboxylic acids (PFCAs), perfluorosulfonic acids (PFSAs)], and their per- and polyfluorinated precursor compounds. Due to global environmental concerns, the manufacture and use of perfluorooctane sulfonic acid (PFOS), perfluorooctanoic acid (PFOA), and perfluorohexanesulfonic acid (PFHxS) have been restricted or phased out and listed to annexes of the United Nations Stockholm Convention of Persistent Organic Pollutants, while some replacement PFAAs, e.g., long-chain PFCAs, are currently undergoing risk assessment for possible listing under the Convention.^4^ Short-chain PFCAs and PFSAs are also currently used as replacements for PFAS,^5^ but their environmental behavior is relatively understudied.

PFAS (mainly PFAAs) have been detected in wild bird species globally, with PFOS generally dominating the measured PFAS profiles in avian species followed by PFOA.^6−8^ Accumulation of PFAAs has been reported in apex predators, like the peregrine falcon (*Falco peregrinus*),^7,9^ which feeds (nearly) exclusively on terrestrial and aquatic birds. PFAS have also been measured in mid-trophic birds like the herring gull (*Larus argentatus*)^10,11^ and songbirds including the tree swallow (*Tachycineta bicolor*)^6,8^ that forage in aquatic–terrestrial habitats. Despite evidence that PFAS are an important class of contaminants of concern for avian wildlife,^6,12^ the potential exposure pathways and the accumulation of major PFAS by wildlife are not fully characterized and understood.

Birds (and other wildlife) may be exposed to PFAS through multiple routes. One route of exposure to PFAS may be through inhalation; the air sacs (“lungs”) of birds are larger than in other species, permitting large volumes of air exchange.^13^ In addition, it is postulated that PFAS are slowly eliminated by respiration in air-breathing organisms due to high protein-air partition coefficients.^14^ Uptake of other contaminants through inhalation, including brominated polycyclic aromatic hydrocarbons and halogenated flame retardants, has been identified as an important route of exposure for birds.^15,16^ Ingestion of freshwater by birds when foraging, drinking, and bathing, may represent another route of exposure to PFAS. We have reported that tree swallows foraging from nearby effluent-receiving waters accumulated higher concentrations of PFAS in their eggs and young chicks.^8^ Finally, diet from aquatic and/or terrestrial sources is considered the major route of exposure to most persistent organic pollutants for biota.^17^

The diet of many species, including tree swallows, can involve invertebrates. Emergent aquatic macroinvertebrates can transfer contaminants from aquatic to terrestrial environments through food web interactions.^18−20^ Aquatic invertebrate larvae develop in the benthic layer of freshwater streams where they can be exposed to contaminants via water, sediment, and plants.^21,22^ During metamorphosis, some contaminants are retained in invertebrate tissues^23^ that, in addition to contaminants accumulated as adults, may be transferred when consumed as prey by predators, such as tree swallows.^24,25^ Although several contaminants, including some PFAS, are known to move from aquatic to terrestrial biota within food webs,^18,26,27^ the emergent macroinvertebrate pathway has not been investigated for the majority of PFAA precursors to date. One study^18^ has investigated the macroinvertebrate pathway for PFOSA and fluorotelomer sulfonate precursors, moving from water to plants and then terrestrial and aquatic invertebrates.

In the present study, we investigated the concentrations, patterns and movement of PFAS in an avian aquatic–terrestrial food web, using the tree swallow as a model species.^6,8,28,29^ Our objectives were to (1) characterize the aquatic–terrestrial food web structure (dietary sources) of tree swallow nestlings; (2) assess the concentrations and profiles of PFAS, including short- and long-chain PFCAs and PFSAs, and precursor compounds, in the abiotic and biotic compartments of the swallows’ food web; (3) calculate bioaccumulation factors (BAFs) and biomagnification factors (BMFs) of individual PFAS in this aquatic–terrestrial food web, and (4) investigate potential relationships of PFAS concentrations in these environmental compartments, specifically among invertebrates, surface water, sediment, and air, with the PFAS concentrations of the tree swallow nestlings.

## Materials and Methods

This study was conducted in accordance with the Canadian Council of Animal Care Guidelines as approved by the Eastern Wildlife Animal Care Committee of Environment and Climate Change Canada (ECCC) (21KF10), and subsequently by the Animal Care Committee of the collaborating institution, McGill University. All necessary scientific permits, and approvals from appropriate authorities, were obtained prior to the study commencing (Canadian Wildlife Service Permit Number: SC-OR-2021-00053).

### Study Site

**Figure 1:**
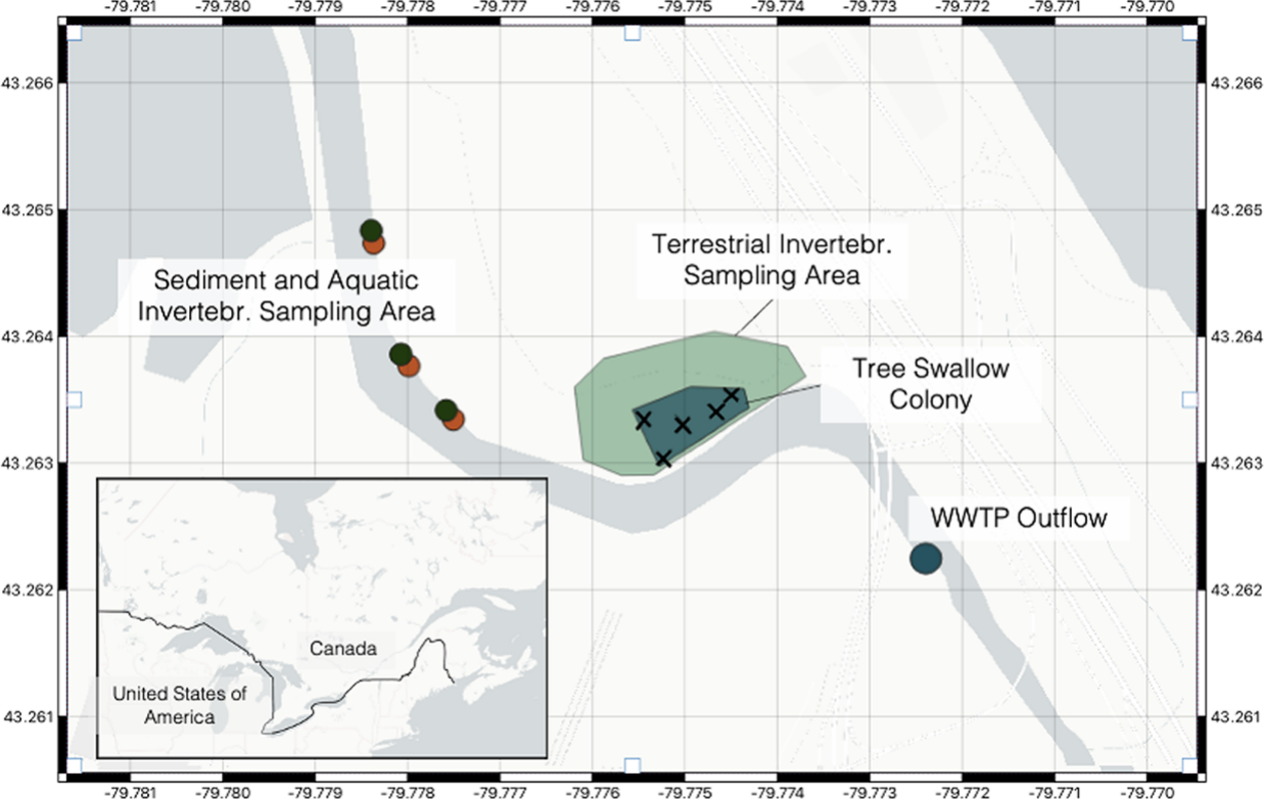
Sampling locations at an established tree swallow colony approximately 250 m from the effluent outflow of a major urban wastewater treatment plant (WWTP, Hamilton) that used secondary treatment processes at the time of the study. Sediment (green circles) and aquatic invertebrate (orange circles) sampling collections occurred 250 m downstream from the tree swallow colony. Air samplers were deployed within the active tree swallow colony (marked by X). Terrestrial invertebrate sampling occurred in the area that tree swallow adults were observed feeding within and surrounding the colony.

Nestlings were collected in 2021 from a long-term breeding colony of tree swallows located in Windermere Basin (43°15′48.3″N 79°46′29.0′′W), Hamilton, Ontario, Canada, within the Laurentian Great Lakes Basin (Figure 1). This colony has 49 nest boxes situated on Red Hill Creek immediately downstream (250 m) of the effluent outflow of the Woodward Wastewater Treatment Plant (WWTP), that used secondary treatment processes at the time of the study. Woodward WWTP is the only WWTP servicing the city of Hamilton, Ontario (2021 population: 570,000).

### Tree Swallow Tissue Collection

Detailed methods for the collection of tree swallow tissues have been described elsewhere^8^ and in the Supporting Information (SI). Briefly, one 10-day-old nestling was randomly selected from each of 15 different nest boxes. These 15 nestlings were euthanized by cervical dislocation and immediately dissected. Thyroid glands were dissected and stored at room temperature for future analyses. Blood, breast muscle, gastrointestinal tracts (GI tracts) including any contents from beak to vent, and livers were collected, blood separated into plasma and red blood cells by centrifugation, and along with the remaining carcass, all were immediately flash frozen in a dry nitrogen shipper. Samples were stored at −80 °C until subsequent analysis.

### Macroinvertebrate Collection

Macroinvertebrates were collected using adapted methods^30−32^ as described in the SI. Briefly, aquatic macroinvertebrates were sampled using six Hester–Dendy (HD) round 14-plate samplers.^32^ Traps were placed in pairs at three different locations, approximately 50 m apart, between 250 and 450 m downstream of the tree swallow colony (Figure 1) and deployed for 4 weeks in duration. Terrestrial macroinvertebrate samples were collected daily for 2 weeks, using sweep nets within the colony and the adjacent surrounding area (∼8000 m^2^) where tree swallows were observed foraging (Hopkins and Fernie, Pers. obs.). Terrestrial macroinvertebrate samples [*n* = 5 each for PFAS and fatty acid (FA) analyses] were identified in the order, and only organisms from the following orders were retained: Hemiptera, Odonata, Diptera, and Ephemeroptera to reflect tree swallow diet. Of the aquatic macroinvertebrate samples, only organisms from the order Amphipoda were sampled as they were the only organisms caught with the HD samplers (*n* = 5 each for PFAS and FAs). Samples were stored at −80 °C until analysis.

### Air, Surface Water, and Sediment Collection

The SI provides full details on the collection methods of passive air samples,^33^ surface water,^18^ and sediment samples. Briefly, five sorbent-impregnated polyurethane foam passive air samplers (SIP-PAS) were deployed at the four corners and the center of the colony (Figure 1) for a 50-day period concurrent with the breeding and nestling development of the tree swallows. Twenty days after nestling tissues were collected, passive air samples were collected and stored individually in amber glass jars at below −10 °C until chemical analysis. Six surface water samples (500 mL) were collected directly from the wastewater effluent outflow pipe in the top 8–10 cm of the water column. Six sediment samples (5 g) were collected 250 m downstream from the three HD trap locations (2 sediment samples/location). Sediment was collected from the top 6 cm of the creek bed. Both surface water and sediment samples were collected 5 days after collecting the tree swallow tissues because of adverse weather in the 5 days immediately following tree swallow collections. The surface water and sediment samples were immediately stored at −20 °C until subsequent analysis.

### Stable Isotope and Fatty Acid Analysis

Stable isotope analysis was conducted using the breast muscle of individual chicks (Ján Veizer Stable Isotope Laboratory, University of Ottawa),^7,29^ and described fully including quality assurance and control (QA/QC) procedures in the SI. In short, lipid-extracted, freeze-dried tree swallow breast muscle powder was loaded into an elemental analyzer, flash combusted, and analyzed on an isotope ratio mass spectrometer. A double-crested cormorant (*Phalacrocorax auritus*) in-house reference material was analyzed with each set of samples, and results were within an acceptable 3% relative percent difference.

The extraction and analysis of fatty acids (FAs) in individual samples of aquatic and terrestrial invertebrates and nestling livers and carcasses, followed Hopkins et al.^8^ and Pedro et al.^34^; see also the SI, which includes related QA/QC procedures. After homogenization and extraction, lipids were *trans-*esterified to their fatty acid methyl ester (FAME) analogues. Isolated FAMEs were reconstituted in hexane and analyzed on an Agilent 8860 gas chromatograph with a flame ionization detector (GC-FID; Agilent Technologies, CA, USA). The average relative percent difference for duplicate samples was 14% for carcasses (*n* = 15), 11% for aquatic macroinvertebrates (*n* = 5), and 21% for terrestrial macroinvertebrates (*n* = 4). Duplicate samples could not be run for liver samples because the mass of individual samples was too small. A US National Institute of Standards and Technology standard reference material (Krill RM 8037) was analyzed concurrently, and results were within 13% of reported values.

### Per- and Polyfluoroalkyl Substance Analysis

Extraction and analysis of ∑_21_PFAS (Table S1) in air SIP-PAS samples were completed by ECCC’s Air Quality Processes and Research Section (Hazardous Air Pollutants laboratory) as previously described.^35^ SIP-PASs were spiked with internal standards (Table S2), extracted, and analyzed for volatile *n*-PFAS using gas chromatography with single quadrupole mass spectrometry (GS-MS), and for ionizable PFAS using ultra-performance liquid chromatography coupled with tandem mass spectrometer (UPLC-MS/MS). Standard recoveries for PFAS were within 70–135% for volatile PFAS, and 55–90% for ionizable PFAS; see the SI for detailed QA/QC procedures. Detection frequencies for the ∑_21_PFAS measured in air samples ranged from 0 to 100%.

Extraction and instrumental analysis of ∑_46_PFAS (Table S1) in surface water, sediment, aquatic invertebrates, terrestrial invertebrates, tree swallow GI tracts and livers was completed by SGS AXYS Analytical (Sidney, British Columbia, Canada) following EPA Method 1633, as previously described.^36^ Samples were spiked with surrogate standards and extracted for identification and quantification of 46 PFAS (see Table S2 for the full list of analytes) by UPLC-MS/MS. Surrogate standard recoveries ranged from 48 to 166% (see Table S4 for instrument detection limit and minimum limit of detection values for PFAS analysis). Detailed methods for PFAS extraction and analysis, and QA/QC procedures, in the environmental compartments can be found in the SI. Detection frequencies for the ∑_46_PFAS measured ranged from 0 to 100% in all compartments (Table S3).

### Statistical Analysis

All statistical analyses were completed using R Studio (2022.07.02.B576).^37^ Of the 66 FAs identified, only seven of the major dietary FAs were analyzed statistically to avoid the influence of minor FAs (those with proportions <0.1%).^8,34^ As FA data are proportional, FA values were arcsine transformed prior to statistical analysis. Only individual PFAS with detectable concentrations in ≥60% of the samples were statistically analyzed (identified in Table S5) and estimated concentrations were calculated for related nondetects (≤40%) using regression probability plotting.^38^ All PFAS concentrations were log-transformed to achieve a normal distribution. Arithmetic means were calculated for duplicate FA and PFAS samples for statistical analysis.

The FA patterns among aquatic and terrestrial invertebrates and tree swallow livers and carcasses were compared using a principal component analysis (PCA) with the *factoextra* and *devtools* packages.^39,40^ A multivariate one-way analysis of variance (MANOVA) was used to test for significant differences in FA patterns among aquatic invertebrates, terrestrial invertebrates, tree swallow liver, and carcass, followed by univariate analysis of variances (ANOVA) and Tukey–Kramer post-hoc tests to examine differences among these compartments for individual FAs.

One-way ANOVAs followed by Tukey–Kramer post hoc analyses were used to test for differences in PFAS concentrations among the abiotic (air, surface water, and sediment) and biotic (aquatic invertebrates, terrestrial invertebrates, and tree swallow GI tracts and livers) compartments. To qualitatively compare PFAS patterns among the abiotic and biotic compartments, we created profiles by calculating the proportion that each congener contributed to the total (∑) PFAS concentration of each sample and then calculated the arithmetic means of the proportions across all samples within each compartment.

Bioaccumulation factors (BAFs) were calculated for individual PFAS congeners that had measurable concentrations in both the abiotic (e.g., air) and biotic (e.g., nestling tree swallows’ GI tracts) compartments. We also calculated non-trophic adjusted biomagnification factors (BMFs) from aquatic and terrestrial invertebrates to tree swallow GI tracts and livers. To account for the varying sample sizes among compartments (reported in Tables 1 and 2), we created new data frames by randomly sampling exact values of individual PFAS congeners from each environmental compartment 1000 times. BAFs and BMFs were then calculated by using the mean concentrations of each PFAS congener from the new data frame. Both BAFs and BMFs were calculated by dividing the mean concentration of the individual PFAS congener in the organism by the concentration of the PFAS congener in the abiotic compartment (air, surface water, or sediment) for BAFs, or by the concentration of the PFAS congener in the prey item of the tree swallow nestlings (aquatic or terrestrial invertebrates) for BMFs.

**Table 1:** Arithmetic Mean Concentrations (*x̅* ± SE; on a Wet Weight Basis in Biotic Samples) of Per and Polyfluoroalkyl Substances (PFAS) Measured in Seven Abiotic and Biotic Environmental Compartments, Air, Surface Water, Sediment, Aquatic Invertebrates, Terrestrial Invertebrates, and Tree Swallow Nestling Gastrointestinal Tracts (GI Tracts) and Livers, Sampled from an Aquatic–Terrestrial Ecosystem in Southwestern Ontario in 2021 (see Figure 1)^a^

	perfluorinated arbon atoms	air (ng/m^3^) *n* = 5		surface water (ng/mL) *n* = 6		sediment (ng/g) *n* = 6		aquatic invertebrates (ng/g) *n* = 5		terrestrial invertebrates (ng/g) *n* = 5		GI tract (ng/g) *n* = 15		liver (ng/g) *n* = 15	
congener	# of perfluorinated carbon atoms	*x̅* ± SE	range	*x̅* ± SE	range	*x̅* ± SE	range	*x̅* ± SE	range	*x̅* ± SE	range	*x̅* ± SE	range	*x̅* ± SE	range
Σ_46_PFAS	NA	1.02 ± 0.07	0.77–1.63	0.14 ± 0.02	0.08–0.43	15.66 ± 2.5	3.16–35.5	13.30 ± 1.14	7.35–21.83	2.47 ± 0.10	2.15–3.35	23.1 ± 0.30	17.7–32.1	67.9 ± 2.03	31.4–132.3
perfluorocarboxylic acids															
PFBA*	C_4_	0.06 ± 0.008	0.04–0.08	0.01 ± <0.001	0.01–0.01	ND	ND	ND	ND	ND	ND	ND	ND	ND	ND
PFPeA*	C_5_	ND	ND	0.01 ± <0.001	0.01–0.01	ND	ND	0.20 ± <0.001	0.19–0.22	ND	ND	ND	ND	ND	ND
PFHxA*	C_6_	0.03 ± 0.01	0.006–0.08	0.02 ± 0.001	0.02–0.02	0.92 ± 0.25	0.36–1.71	0.23 ± 0.04	0.16–0.36	ND	ND	ND	ND	ND	ND
PFHpA*	C_7_	0.002 ± <0.001	0.001–0.004	0.004 ± <0.001	0.003–0.004	ND	ND	ND	ND	ND	ND	ND	ND	ND	ND
PFOA*	C_8_	0.007 ± 0.001	0.005–0.01	0.006 ± <0.001	0.006–0.006	0.29 ± 0.10	0.11–0.53	0.10 ± <0.001	0.10–0.11	ND	ND	0.12 ± <0.001	0.10–0.15	ND	ND
PFNA*	C_9_	0.001 ± <0.001	0.001–0.002	0.001 ± <0.001	0.001–0.001	ND	ND	ND	ND	ND	ND	0.37 ± 0.02	0.12–0.61	1.34 ± 0.07	1.02–1.54
PFDA*	C_10_	0.002 ± <0.001	0.002–0.004	0.001 ± <0.001	0.001–0.002	0.44 ± 0.11	0.13–0.76	0.1 ± <0.001	0.10	ND	ND	0.82 ± 0.06	0.44–1.35	2.7 ± 0.15	1.78–4.09
PFUnA*	C_11_	ND	ND	ND	ND	ND	ND	ND	ND	ND	ND	0.35 ± 0.02	0.25–0.49	0.87 ± 0.04	0.72–1.23
PFDoA*	C_12_	ND	ND	ND	ND	0.65 ± 0.15	0.21–1.02	0.40 ± 0.02	0.35–0.46	ND	ND	0.19 ± 0.01	0.14–0.25	ND	ND
PFTrDA*	C_13_	0.002 ± <0.001	0.002–0.002	ND	ND	0.28 ± 0.07	0.10–0.49	0.24 ± <0.001	0.22–0.26	ND	ND	0.17 ± <0.001	0.12–0.24	ND	ND
PFTeDA*	C_14_	ND	ND	ND	ND	0.47 ± 0.13	0.13–0.91	0.27 ± 0.04	0.20–0.42	ND	ND	0.18 ± 0.01	0.14–0.32	ND	ND
perfluorosulfonic acids															
PFBS*	C_4_	0.004 ± <0.001	0.002–0.005	0.004 ± <0.001	0.003–0.004	ND	ND	ND	ND	ND	ND	ND	ND	ND	ND
PFPeS*	C_5_	X	X	5.0 × 10^–4^ ± <0.001	4.0 × 10^–4^ − 5.0 × 10^–4^	ND	ND	ND	ND	ND	ND	ND	ND	ND	ND
PFHxS*	C_6_	0.001 ± <0.001	7.0 × 10^4^ − 0.003	0.002 ± <0.001	0.002–0.002	ND	ND	ND	ND	ND	ND	ND	ND	ND	ND
PFOS*	C_8_	0.10 ± 0.04	0.03–0.26	0.006 ± <0.001	0.005–0.007	1.99 ± 0.57	0.51–4.4	1.46 ± 0.07	1.28–1.57	2.08 ± 0.19	1.90–2.83	9.6 ± 0.58	6.0–14.9	29.18 ± 1.68	21.8–49.1
PFDS*	C_10_	ND	ND	ND	ND	0.51 ± 0.09	0.25–0.56	0.12 ± <0.001	0.11–0.12	ND	ND	0.17 ± <0.001	0.12–0.21	ND	ND
fluorotelomer sulfonates															
6:2 FTS*	C_6_	0.003 ± <0.001	0.003–0.005	0.006 ± <0.001	0.006–0.006	ND	ND	ND	ND	0.41 ± 0.04	0.36–0.52	ND	ND	ND	ND
8:2 FTS*	C_8_	0.001 ± <0.001	2.0 × 10^–4^−3.0 × 10^–4^	ND	ND	ND	ND	ND	ND	ND	ND	ND	ND	ND	ND
10:2 FTS*	C_10_	0.002 ± <0.001	1.0 × 10^4^ − 0.001	X	X	X	X	X	X	X	X	X	X	X	X
fluorotelomer alcohols															
6:2 FTOH*	C_6_	0.33 ± 0.05	0.24–0.51	X	X	X	X	X	X	X	X	X	X	X	X
8:2 FTOH*	C_8_	0.28 ± 0.04	0.20–0.43	X	X	X	X	X	X	X	X	X	X	X	X
10:2 FTOH*	C_10_	0.19 ± 0.02	0.13–0.24	X	X	X	X	X	X	X	X	X	X	X	X
fluorotelomer carboxylates															
5:3 FTCA*	C_5_	X	X	ND	ND	ND	ND	7.59 ± 2.23	3.82–15.9	ND	ND	4.06 ± 0.31	2.25–5.85	ND	ND
7:3 FTCA*	C_7_	X	X	ND	ND	ND	ND	ND	ND	ND	ND	4.57 ± 0.35	2.24–8.03	24.68 ± 3.13	11.8–43.1
perfluorooctane sulfonamides															
PFOSA*	C_8_	X	X	ND	ND	0.35 ± 0.10	0.11–0.61	0.33 ± 0.03	0.26–0.43	ND	ND	0.13 ± <0.001	0.10–0.15	ND	ND
N-MeFOSA*	C_8_	0.002 ± <0.001	0.001–0.004	ND	ND	ND	ND	ND	ND	ND	ND	0.65 ± 0.09	0.15–1.46	ND	ND
N-EtFOSA*	C_8_	0.002 ± <0.001	0.001–0.003	ND	ND	ND	ND	ND	ND	ND	ND	0.44 ± 0.05	0.21–0.80	ND	ND
perfluorooctane sulfonamido acetic acids															
N-MeFOSAA*	C_8_	X	X	0.06 ± 0.06	5.0 × 10^–4^ − 0.36	2.05 ± 0.20	0.55–1.7	0.48 ± 0.10	0.25–0.81	ND	ND	0.30 ± 0.01	0.23–0.42	0.74 ± 0.04	0.57–0.96
N-EtFOSAA*	C_8_	X	X	7.0 × 10^–4^ ± <0.001	5.0 × 10^–4^ − 8.0 × 10^–4^	1.66 ± 0.37	0.65–3.22	0.78 ± 0.24	0.34–1.67	ND	ND	0.32 ± 0.02	0.19–0.47	0.79 ± 0.05	0.61–0.96
perfluorooctane sulfonamide ethanols															
N-MeFOSE*	C_8_	0.009 ± 0.002	0.006–0.02	ND	ND	ND	ND	ND	ND	ND	ND	ND	ND	ND	ND
N-EtFOSE*	C_8_	0.005 ± 0.002	0.004–0.006	ND	ND	ND	ND	ND	ND	ND	ND	3.11 ± 1.27	0.63–2.81	ND	ND

a See Table S1 for full PFAS names. “X” indicates that a specific PFAS congener was not determined in that compartment, and “ND” means that the PFAS concentration was below the detection limit.

**Table 2:** Calculated Bioaccumulation (BAFs) and Biomagnification Factors (BMFs) for Per and Polyfluoroalkyl Substances (PFAS) in Abiotic and Biotic Compartments of a Tree Swallow Food Web in Southwestern Ontario in 2021^a^

short-chain PFCAs				long-chain PFCAs					long-chain PFSAs		precursors							
		PFPeA	PFHxA	PFOA	PFNA	PFDA	PFDoA	PFTrDA	PFOS	PFDS	6:2 FTS	5:3 FTCA	PFOSA	N-MeFOSA	N-EtFOSA	N-MeFOSAA	N-EtFOSAA	N-EtFOSE
BAF	AI/SW	20.1	11.5	16.7	-	100	-	-	243	-	-	-	-	-	-	7.98	1114	-
	AI/sediment	-	0.25	0.34	-	-	0.62	0.86	0.73	0.24	-	-	0.94	-	-	0.23	0.47	-
	GI/SW	-	-	-	370	820	-	-	1600	-	-	-	-	-	-	5.11	457	-
	liver/SW	-	-	-	1340	-	-	-	4863	-	-	-	-	-	-	12.3	1128	-
	TI/air	-	-	-	-	-	-	-	20.8	-	137	-	-	-	-	-	-	-
	GI/air	-	-	-	370	410	-	85.2	95.8	-	-	-	-	325	220	-	-	622
	liver/air	-	-	-	1340	1350	-	-	292	-	-	-	-	-	-	-	-	-
BMF	GI/AI	-	-	-	-	8.21	0.48	0.71	6.58	1.42	-	0.53	0.39	-	-	0.63	0.41	-
	liver/AI	-	-	-	-	27.3	-	-	19.9	-	-	-	-	-	-	1.54	1.01	-
	GI/TI	-	-	-	-	-	-	-	4.62	-	-	-	-	-	-	-	-	-
	liver/TI	-	-	-	-	-	-	-	14.0	-	-	-	-	-	-	-	-	-

a Short-chain PFCAs: ≤ C_7_. Long-chain PFCAs: ≥ C_8_. Long-chain PFSAs: ≥ C_7_. See Table S1 for full PFAS chemical names· Nestling samples involved their gastrointestinal tract (GI) and livers, SW = surface water, TI= terrestrial invertebrates, and AI = aquatic invertebrates. BAFs and BMFs that could not be calculated are identified as “-”·

We used multiple factor analysis (MFA) to compare the PFAS patterns and visually assess the potential influence of the abiotic and biotic compartments on tree swallow nestling tissue PFAS concentrations. The MFA organizes groups of variables similar in nature, and produces a weight for each group, which then balances the influence of the groups.^41^ Variables or PFAS congeners were grouped individually, and individuals were grouped by environmental compartment (air, surface water, sediment, aquatic invertebrates, terrestrial invertebrates, and swallow nestling GI tracts and livers). Means and standard errors (±SEM) are presented, and *p*-values ≤0.05 were considered statistically significant.

## Results and Discussion

### Food Web Structure and Feeding Patterns of Tree Swallow Nestlings

**Figure 2:**
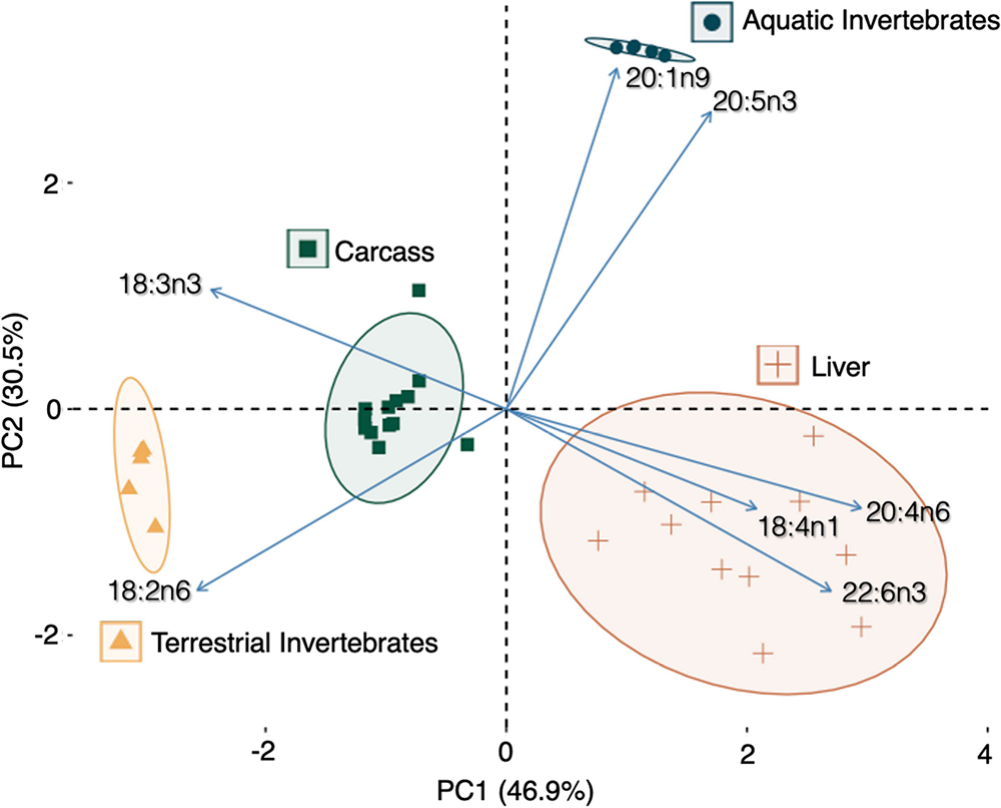
Principal component analysis of fatty acid signatures (arcine-square root transformed, scaled) of aquatic and terrestrial invertebrates, and the carcasses and livers of nestling tree swallows collected in the Laurentian Great Lakes Basin in southwestern Ontario in 2021. Ellipses represent the 95% confidence intervals.

Stable isotopes (breast muscle) and FA signatures (liver, carcass) measured in the same tree swallow nestlings indicated a diet consisting of both terrestrial and aquatic food resources. Muscle δ^13^C (−23.3 ± 0.12‰) and δ^15^N (12.7 ± 0.14‰) were consistent with previous stable isotope values measured in red blood cells of swallow nestlings at this study site, and this range of carbon SI values suggests that their diet continues to consist more of terrestrial than aquatic macroinvertebrates.^8^ The results of PCA based on the FA signatures of the chicks’ food web are consistent with these findings (Figure 2). The aquatic invertebrates loaded positively on both PC1 (accounting for 47% of the FA variation) and PC2 (31% of the variation) and were associated with the FAs, 20:1n9 and 20:5n3 (Figure 2), which are indicative of aquatic ecosystems.^42^ The FAs of the terrestrial invertebrates loaded negatively on PC1 and PC2 and were associated with the terrestrial-based FA, 18:2n6.^42,43^ For the swallow chicks, the carcass FA values, although not the hepatic FA values, clustered between the two invertebrate groups yet were closer to the terrestrial invertebrates, particularly along PC2. The duality of dietary sources for the tree swallow chicks is further substantiated by the significant differences in FA signatures among the aquatic invertebrates, terrestrial invertebrates, tree swallow livers, and nestling carcasses (Pillai’s Trace = 2.89, F_3,33_ = 14.63, *p* < 0.001; Table S6 and Figure S1). Proportions of the terrestrial FA, 18:2n6, in descending rank order, were higher in terrestrial invertebrates > nestling carcasses > nestling livers > aquatic invertebrates (F_4,29_ = 65.73, all *p* < 0.001) (Figure S1). The proportions of the aquatic-associated FA, 20:5n3, were higher in aquatic invertebrates (F_4,29_ = 38.88, *p* < 0.001) than in other compartments, but similar among terrestrial invertebrates, nestling livers, and nestling carcasses (all *p* ≥ 0.20). Carcass profiles are closely aligned with the profiles of both dietary sources, aquatic and terrestrial macroinvertebrates. Hepatic FA patterns were more distinct, likely as liver FA profiles are highly influenced by metabolism, as the liver is the central organ of FA metabolism.^44^ Collectively, the SI and FA results indicate that the diet of the nestling tree swallows, and thus potentially exposure to PFAS, encompasses the terrestrial and aquatic compartments of their food web.

### PFAS Concentrations in Abiotic and Biotic Compartments

**Figure 3:**
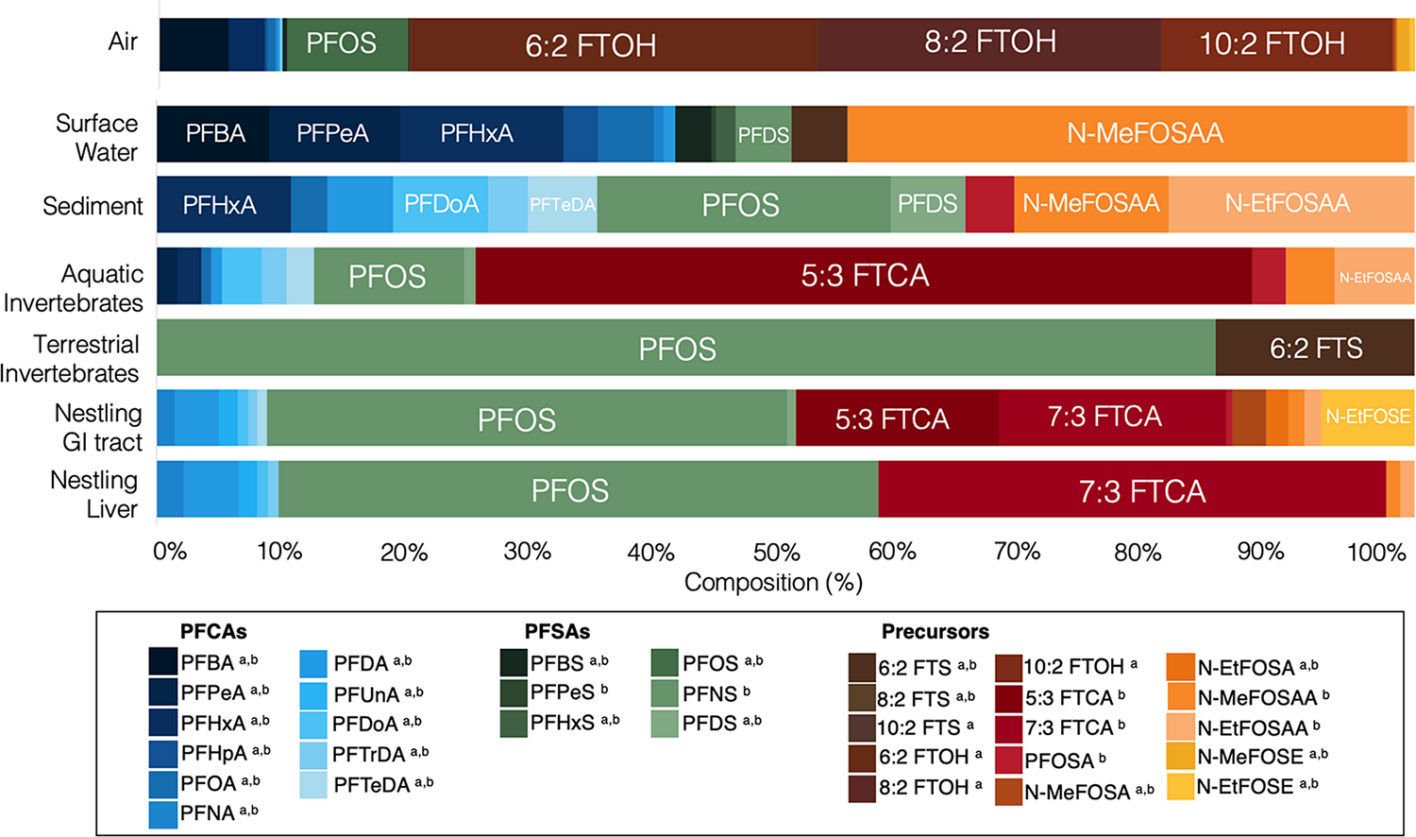
Profiles of perfluoroalkyl substances (% of ∑PFAS concentrations) in the abiotic and biotic compartments of the food web of tree swallow samples collected in 2021 near the outfall of a major urban wastewater treatment plant. Across all compartments including air, all 11 selected PFCAs were measured, as were four of six PFSAs, and six of 15 precursors. GI tracts: nestling gastrointestinal tract including any contents. Compounds analyzed in air denoted by a, and compounds analyzed in surface water, sediment, aquatic and terrestrial invertebrates, and tree swallow GI tracts and livers are denoted by b.

There were distinct differences in the PFAS profiles among the abiotic and biotic compartments of the tree swallow food web (Figure 3). PFOS was the dominant congener of the terrestrial invertebrates (84%), nestling livers (48%), and GI tracts (41%), whereas comparatively, PFOS contributed less to the overall PFAS profiles of the aquatic invertebrates (12%), sediment (23%), surface water (4%), and air (10%). There were relatively minor contributions of the remaining measured PFSAs to the overall PFAS profiles of the abiotic and biotic compartments of the food web. The dominance of PFOS in avian PFAS profiles is well documented^6−8,45^ and expected due to its environmental persistence and the ongoing disposal of (consumer) products containing historical PFOS.^46^

The short-chain C_4_–C_7_ PFCAs (i.e., PFBA, PFPeA, PFHxA, PFHpA) represented approximately 66% of the overall PFAS profile of surface water, whereas collectively, short- and long-chain PFCAs contributed less than 15% to the PFAS profiles of air, sediment, invertebrates, and chicks (Figure 3). Short-chain PFCAs (i.e., PFBA, PFPeA, PFHxA) preferentially remain in their dissolved state and partition into water, unlike the longer-chain PFCAs that partition more into sediment and biota.^47^ In addition to the dominance of short-chain PFCAs, the sediment profile also saw large contributions of the two perfluorooctane sulfonamidoacetic acids (FOSAAs), MeFOSAA (12%), and EtFOSAA (20%).

The PFAS profiles of the aquatic invertebrates and air were dominated by the PFAA precursor compounds measured in this study. The fluorotelomer carboxylate, 5:3 FTCA, was the dominant PFAS congener in the aquatic invertebrates, and together 5:3 FTCA and 7:3 FTCA comprised approximately 40% of the total PFAS profile of the tree swallow tissues, suggesting that aquatic invertebrates may be an important source of these carboxylates for the tree swallow chicks. The fluorotelomer alcohols, 6:2 FTOH, 8:2 FTOH, and 10:2 FTOH, comprised almost 80% of, and thus dominated, the air PFAS profile but were not analyzed in the tree swallow chicks or other food web compartments because appropriate analytical methods were not developed for such samples at the time of the present study. Since fluorotelomer alcohols are rapidly transformed in the environment, and by metabolism within biota,^48^ we hypothesize that, if analyzed, they would not be detectable in the nestlings because of a lack of bioavailability or rapid metabolism by the chicks. Additional research is required to test this hypothesis. The precursor compound and known substitute of PFOS, 6:2 FTS, comprised 27% of the PFAS profile of the terrestrial invertebrates but was undetected in the tree swallow chicks and other biotic food web compartments, suggesting either the lack of bioavailability to vertebrate species or potentially rapid biotransformation to other PFAS by predators like the swallows. 6:2 FTS is known to degrade into intermediate metabolites such as PFHpA, PFHxA, PFPeA, and PFBA.^49^ Although MeFOSAA was the largest component of the PFAS profile in surface water (44%), it contributed to ≤ 4% of the profiles of the remaining abiotic and biotic compartments, suggesting it may also be biotransformed into further biotransformation products like PFOA^50^ by biota.

Concentrations of individual PFAS varied among the food web compartments in this study. Notably PFOS, the three long-chain PFCAs, perfluorononanoic acid (PFNA), perfluorodecanoic acid (PFDA), and PFUnA, and the precursor, 7:3 FTCA (F ≥ 7.92, all *p* < 0.001), showed the highest concentrations in the nestling livers (all *p* ≤ 0.01), followed by nestling GI tracts (all *p* ≤ 0.02), with lower and more variable concentrations in the remaining biotic and abiotic compartments (Tables 2 and S7). These results are consistent with previously reported PFAS concentration patterns in birds.^7,8^ PFAS tissue distribution patterns, including the sequestering of the highest concentrations of PFAS in the liver, were similarly documented in previous studies with tree swallows at this study site in 2019^8^ and in Michigan, United States,^6^ as well as in Great tits (*Parus major*) in Antwerp, Belgium^51^ and in higher trophic level birds like the peregrine falcon (*Falco peregrinus*)^7^ in southern Ontario, Canada. Concentrations of PFDoA, perfluorotridecanoic acid (PFTrDA), PFTeDA, PFDS, and the two precursors, N-MeFOSAA and N-EtFOSAA, were different among abiotic and biotic compartments (*F* ≥ 14.52, all *p* < 0.001), where the highest concentration was in sediment, followed by either the aquatic invertebrates or nestling tissues (all *p* ≤ 0.001). Additionally, higher concentrations of N-MeFOSA and N-EtFOSA were measured in the nestling GI tracts than in the air samples (both *p* < 0.001). All remaining PFAS were either similar among abiotic and biotic compartments, or only detected in one compartment of this food web. Another PFAS riparian food web study performed in Sweden,^18^ similarly reported that biotic compartments had significantly greater ∑_24_PFAS concentrations than abiotic compartments. However, PFAS concentrations across all of the compartments in the study conducted in Sweden were higher than those in the present study, possibly due to the proximity of their sampling location to a military airport. Concentrations of ∑_46_PFAS and individual congeners in surface water effluent in the current study were greater than previously reported effluent receiving surface water in Berlin, Germany (∑_10_PFAS),^52^ which may be attributed to differences in WWTP inputs or treatments. Concentrations of individual PFAS congeners in air samples, and the overall dominance of FTOHs compared to other PFAS congeners, in the present study, were largely consistent with reported concentrations of the same individual PFAS and dominance of FTOHs in urban air samples collected globally.^53^ To our knowledge, the current study is the first to comprehensively characterize not only PFAAs, but also the following precursor subgroups; fluorotelomer sulfonates, fluorotelomer carboxylates, perfluorooctane sulfonamides, FOSAAs, and the perfluorooctane sulfonamide ethanols across multiple abiotic and biotic compartments.

### Bioaccumulation and Biomagnification of PFAS in the Aquatic–Terrestrial Food Web

Calculated BAFs represent the bioaccumulative potential of PFAS in biota from the surrounding environment and were mostly far greater than 1 (Table 2), indicating bioconcentration or bioaccumulation of many individual PFAS congeners from water and air. The only exception to this was BAFs less than 1 for all PFAS congeners for aquatic invertebrates from sediment. Calculated BAFs from air to terrestrial invertebrates, nestling GI tracts and nestling livers, were all >1 (20.8–1350), suggesting inhalation as an important route of exposure and accumulation of these PFAS congeners for these birds. Consistent with a previous study,^18^ PFOS generally showed the highest bioaccumulation of all the measured PFAS congeners (0.73–4863). After PFOS, the long-chain PFCAs, PFNA (370–1340), and PFDA (100–1350), were most bioaccumulative, followed by phased-out congener PFOA (0.34–16.7), whereas short-chain and other long-chain PFCAs bioaccumulated less or not at all. Chain length is considered to have a strong influence on the bioaccumulative potential of PFAS, as PFAA bioaccumulation increases until around 11–12 carbon chain length.^54−56^ Nevertheless, in the present study, the long-chain PFSA, PFDS, did not bioaccumulate based on the calculated BAFs, although a BAF could not be calculated from sediment to aquatic invertebrates. This contrasts with PFOS, the other long-chain PFSA examined here. In addition, there was no evidence of the bioaccumulation of PFDS from the abiotic compartments to the tree swallows. These results, which suggest that PFDS does not bioaccumulate in biota, require additional investigations, as bioaccumulation of PFDS in biota was reported in a riparian food web in Sweden.^18^ We hypothesize that this difference may be due to prey selection in our study or the possible metabolism of PFDS by higher trophic level predators in our study (swallows) compared to lower trophic level prey in the Swedish study (spiders). We observed bioaccumulation values for PFOS, PFDA, and PFNA that were similar to those calculated in the riparian food web in Sweden,^18^ where calculated values were >1000 in both studies. The precursor PFAS compounds, N-MeFOSA, N-EtFOSA, N-MeFOSAA, N-EtFOSE, and especially N-EtFOSAA were (highly) bioaccumulative (BAFs 12.3–1128), in the food web of the present study, with some values being similar to the BAFs for PFOS (Table 2); the exceptions were the BAFs (<1) for sediment to aquatic invertebrates. In the present study, N-EtFOSE was highly bioaccumulative from air to tree swallow GI tracts (622), suggesting bioaccumulative properties, whereas a previous study concluded that N-EtFOSE did not bioaccumulate from soils.^57^ Given the limited number of studies investigating calculated BAFs for PFAS precursors, further studies are recommended. To our knowledge, our study is the first to consider bioaccumulation of many PFAS precursors, including the understudied perfluorooctane sulfonamides, FOSAAs, and perfluorooctanesulfonamide ethanols in an aquatic–terrestrial environment and suggests that some replacement PFAS may show similar bioaccumulation behavior to those PFAS that are now banned or regulated.

PFOS was found to biomagnify through the aquatic–terrestrial food web of tree swallows in the present study, with calculated BMFs > 1 (4.62–19.9) for all comparisons (Table 2). These calculated BMFs for PFOS in the present aquatic–terrestrial food web are much greater than those previously reported for PFOS in a piscivorous food web in Lake Ontario^58^ and may reflect the influence of the nearby WWTP as a localized source of PFOS in the food web of the present tree swallows within a small geographical area, compared to the diffuse sources of PFOS in the piscivorous food web of Lake Ontario, a much larger area. In the present study, PFDA was the only other PFAS found to biomagnify (8.21–27.3), and to a similar extent as PFOS, while PFNA biomagnification could not be calculated and PFDoA and PFTrDA BMFs were <1. Both PFOS and PFDA have been found to biomagnify in other food webs.^17,59^ Despite some being highly bioaccumulative, the PFAS precursors did not biomagnify with the exception of the weak biomagnification of N-MeFOSAA and N-EtFOSAA from aquatic invertebrates to chick livers (1.0–1.5). Although 5:3 FTCA was detected at high concentrations in aquatic invertebrates and nestling GI tracts, it was not found to biomagnify (<1.0). It is possible that the consumption of aquatic invertebrates is not the source of 5:3 FTCA for the chicks, but instead, it is from the degradation of N-MeFOSAA and N-EtFOSAA precursors in the chicks. Despite high calculated BAFs for the PFAS precursors, it is possible that BMFs are low due to the biotransformation or metabolism of these precursors. For example, Et-PFOSA and PFOSA are known to biotransform to PFOS in rainbow trout (*Oncorhynchus mykiss*),^60^ however, the biotransformation of PFCA precursors has not yet been investigated.

### Influence of Environmental Compartments on Tree Swallow PFAS Exposure

**Figure 4:**
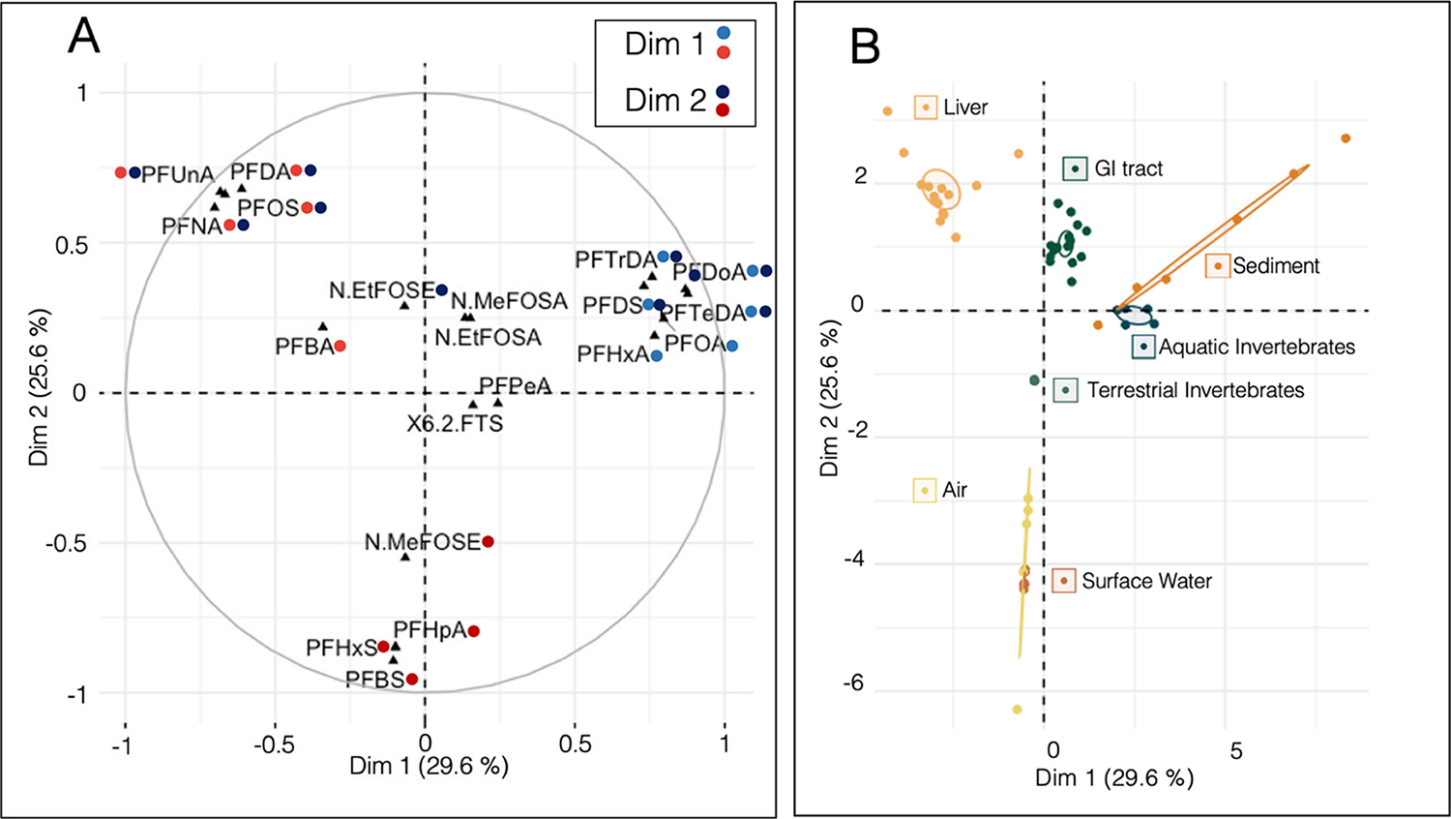
Comparisons of the distribution of perfluoroalkyl substances (PFAS) in abiotic and biotic compartments of a tree swallow food web in the Laurentian Great Lakes Basin in southwestern Ontario in 2021, a multiple factor analysis (MFA). Panel A identifies the loading of the variables (PFAS congeners) to the first two MFA dimensions, variables significantly correlated to a dimension are colored blue (positive) or red (negative). Panel B identifies the loading of the individuals to the first two MFA dimensions. PFAS concentrations were log-transformed and scaled, and ellipses represent 95% confidence intervals. See Table S8 for variable correlations to the first two dimensions.

While the results of the MFA should be considered exploratory and require future research, distinct differences were evident in the distribution of PFAS congeners among the abiotic and biotic compartments of the tree swallow food web using MFA (Figure 4A,B, see Table S9 for variable correlations to each dimension). Nestling GI tracts clustered intermediately between the terrestrial invertebrates and aquatic invertebrates (Figure 4B), and consequently, it is likely that the PFAS concentrations in the swallows’ GI tracts are influenced by the PFAS concentrations measured in the terrestrial and aquatic invertebrate compartments. This observed pattern is consistent with the tree swallow dietary patterns presented earlier, where FA signatures and carbon SIs identified that the chicks consumed both terrestrial and aquatic invertebrates. Tree swallow nestling livers were separated from the other abiotic and biotic compartments (Figure 4B), and loaded toward PFOS, and long-chain PFCAs, PFNA, PFDA, and PFUnA (Figure 4A). Nestling livers had the highest concentrations of PFAS of all measured compartments in this study, and some long-chain PFCAs are known to preferentially accumulate in protein-rich tissues such as livers.^61^ Despite the high BAFs for air to biota presented earlier, there was no overlap between the PFAS concentrations in air and in tree swallow nestlings (Figure 4B). The lack of overlap between air and nestling PFAS concentrations in the MFA may be due to the presence of short-chain PFAAs in air samples but not nestlings. An alternative explanation is that most of the MFA variation was driven by the high concentrations of PFOS and long-chain PFCAs, not the precursors that are comparatively minor despite high bioaccumulation potential from air to swallows. Nevertheless, inhalation of PFAS is still a (likely) source of PFAS for tree swallows, as inhalation of PFAS is well documented for humans,^62^ and for birds inhaling polycyclic aromatic hydrocarbons^15^ and halogenated flame retardants.^16^ Future research should further investigate inhalation as a route of exposure to PFAS for birds by measuring PFAS concentrations, specifically in lung tissue only.

Our study demonstrates that tree swallows within an aquatic and terrestrial food web are exposed to bioaccumulative PFAS and most precursors through the consumption of aquatic and terrestrial invertebrates. Inhalation from air for certain PFAS precursors is also likely given the high bioaccumulation potential found from air to nestlings. Aerial deposition of PFAS on feathers and fur and thus preening and grooming should also be investigated as an additional route of exposure to PFAS for wildlife. Our results show that the long-chain PFCAs (i.e., PFDA, PFNA, PFDoA) were present and appeared to bioaccumulate in most of the biotic compartments of this riparian food web. Aquatic–terrestrial food webs in, for example, wetlands, provide important food sources and habitat for many wildlife species, including multiple insectivores (e.g., birds, bats, small mammals, amphibians) that may thus also bioaccumulate long-chain PFCAs and other PFAS. These findings are important for the current proposed listing of long-chain PFCAs to the U.N. Stockholm Convention on Persistent Organic Pollutants.^4^

## Data Availability

The data underlying this study are available in the published article and its Supporting Information. Data on PFAS concentrations in the gastrointestinal tract contents generated during this study are available as a USGS data release.^63^

## Supporting Information Available

Collection methods: tree swallow tissues, terrestrial and aquatic macroinvertebrates, air, surface water, and surficial sediment, extraction and analysis of dietary tracers, extraction and analysis of per- and polyfluoroalkyl substances (PFAS), fatty acid comparisons among biota, PFAS comparisons among environmental compartments, bioaccumulation and biomagnification factor calculation standard deviations, multiple factor analysis correlations, and raw data results for stable isotopes, fatty acids, and PFAS (PDF)
