# Supplementary material for: Characterizing the Movement of Per- and Polyfluoroalkyl Substances in an Avian Aquatic–Terrestrial Food Web
Source: Environ Sci Technol. 2023 Nov 24;57(48):20249–60. doi: 10.1021/acs.est.3c06944 (PMC10702434; doi:10.1021/acs.est.3c06944)
Supplement: Supplementary file 1 — es3c06944_si_001.pdf [file es3c06944_si_001.pdf]

Supporting Information

Characterizing the Movement of Per- and Polyfluoroalkyl Substances in an Avian Aquatic-Terrestrial Food Web

Kailee E. Hopkins[1,2]*, Melissa A. McKinney[2], Amandeep Saini[3], Robert J. Letcher[4], Natalie K. Karouna-Renier[5], Kim J. Fernie[1,2]

[1]Ecotoxicology and Wildlife Health Division, Environment and Climate Change Canada, 867 Lakeshore Road, Burlington, ON, L7R 4A6, Canada

[2]Department of Natural Resource Sciences, McGill University, 21111 Lakeshore Road, Sainte-Anne-de-Bellevue, QC, H9X 3V9, Canada

[3]Air Quality Processes Research Section, Environment and Climate Change Canada, 4905 Dufferin Street, North York ON, M3H 5T4, Canada

[4]Ecotoxicology and Wildlife Health Division, Environment and Climate Change Canada, Carleton University, 1125 Colonel By Drive, Ottawa, ON, K1A 0H3, Canada

[5]U.S. Geological Survey, Eastern Ecological Science Center, Patuxent Research Refuge, 12302 Beech Forest Road, Laurel, MD, 20708, United States

Pages: 36

Tables: 12

Figures: 1

Any use of trade, product, or firm names is for descriptive purposes only and does not imply endorsement by the U.S. Government.

TABLE OF CONTENTS

Materials and Methods 5

Tree Swallow Tissue Collection...5

Macroinvertebrate Collection 5

Air, Water and Sediment Collection 6

Stable Isotope and Fatty Acid analysis...7

PFAS Analysis 8

Results...9

PFAS Detection Frequencies...9

References..35

Table S1- Subclass, number of perfluorinated carbons, and acronym of 46 target per- and polyfluoroalkyl substances analyzed in environmental compartments (air, surface water, sediment, aquatic and terrestrial invertebrates, and tree swallow gastrointestinal tracts and livers). Compounds analyzed in air denoted by [a], and compounds measured in surface water, sediment, aquatic and terrestrial invertebrates, and tree swallow gastrointestinal tracts and livers are denoted by [b]...11

Table S2 - Surrogate standards for perfluoroalkyl substance (PFAS) extraction and analysis in environmental samples collected from an aquatic-terrestrial food web in the Laurentian Great Lakes Basin in southwestern Ontario, Canada. See Table S1 for full PFAS names. ..12

Table S3 - Detection frequencies (%) of perfluorocarboxylic acids (PFCAs), perfluorosulfonic acids (PFSAs), and) precursors sampled in air, surface water, sediment, aquatic invertebrates, terrestrial invertebrates, and tree swallow nestling GI tracts and livers near a wastewater treatment plant site in southwestern Ontario, Canada (See Figure 1). Congeners not analyzed within a given compartment are denoted with an X. See Table S2 for full PFAS chemical names. 13

Table S4- Instrument limit of detection (IDL) in air samples and minimum limit of detection (MLOD) in surface water, sediment, aquatic invertebrates, terrestrial invertebrates, and tree swallow nestling gastrointestinal tracts (GI tracts) and livers for 46 per- and polyfluoroalkyl substances. Congeners not analyzed within a given compartment are denoted with an X See Table S1 for full PFAS names. 14

Table S5 - Perfluoroalkyl substances (PFAS) that were detected in ≥ 60 % of samples and therefore included in subsequent statistical analyses. Checks indicate congeners ≥ 60 % detection frequency in the respective compartment, NA indicates ≤ 60 % detection frequency, or the congener wasn't analyzed...15

Table S6 - A comparison of five dietary fatty acids from aquatic invertebrates (AI), terrestrial invertebrates (TI), and tissues of tree swallow nestlings (i.e., livers, carcasses). Means ± SEMs are presented, along with the results of Pearson/Spearman's correlations of fatty acids with the principal components generated by the principal component analysis (PCA) of FA proportions of these biota. See Fig. 2 for PCA biplot. 16

Table S7 - Comparisons of perfluoroalkyl substance (PFAS) concentrations among abiotic and biotic compartments of a tree swallow food web. PFAS concentrations of terrestrial insects

were excluded from these statistical comparisons except for PFOS and 6:2 FTS, since the remaining PFAS congeners were undetected (Table S4). Surface water (water), sediment (S), aquatic invertebrates (AI), terrestrial invertebrates (TI), nestling gastrointestinal tracts (GIT), nestling livers. See Table S1 for full PFAS names. 17
Table S8 – Standard deviations of calculated bioaccumulation factors (BAFs) and biomagnification factors (BMFs) for per- and polyfluoroalkyl substances (PFAS) in abiotic and biotic compartments of a tree swallow food web in southwestern Ontario in 2021. Nestling samples involved their gastrointestinal tract and livers. 18
Table S9 – Correlations of the first two dimensions of a multiple factor analysis (MFA) with the concentrations of individual PFAS congeners and precursors measured in abiotic (i.e., air, surface water, sediment) and biotic (i.e., aquatic and terrestrial macroinvertebrates, tree swallow nestling gastrointestinal tracts and livers) samples collected from an aquatic-terrestrial food web in southwestern Ontario. See Fig. 4 for the MFA variables and statistical results. 19
Table S10 – Values of stable carbon ($\delta^{13}C$) and nitrogen ($\delta^{15}N$) isotopes for all tree swallow nestling breast muscle samples collected in 2021 from an aquatic-terrestrial ecosystem in southwestern Ontario. 21
Table S11 – Proportions of individual fatty acids relative to the sum of the 66 fatty acids quantified in aquatic invertebrate, terrestrial invertebrate, and tree swallow liver and carcass samples collected in 2021 from an aquatic-terrestrial food web in southwestern Ontario....22
Table S12 - Concentrations (ng/g in air, ng/mL in surface water and rinsate, ng/g dw in sediment, and ng/g ww in biota samples) of 46 per- and polyfluoroalkyl substances identified in passive air, surface water, sediment, aquatic invertebrates, terrestrial invertebrates, and tree swallow GI-Tracts and livers collected from an aquatic-terrestrial food web. 30

Fig. S1 - Profiles of five selected dietary fatty acid (FA) proportions (% of ∑FA proportions) measured in aquatic invertebrates and terrestrial invertebrates, and in tree swallow nestling livers and carcasses. Letters denote significant differences in proportions between sample types (all $p \leq 0.05$). Only the major dietary FAs, those with proportions greater than 0.1 % were examined, and we selected the top 5 major FAs in the tree swallow nestling carcass for statistical analysis. 20

Materials and Methods

Tree Swallow Tissue Collection

Reproductive monitoring was conducted during the breeding season, April through June, to determine a reproductive timeline (e.g., clutch initiation date, incubation start date, and estimated hatching date). Fifteen chicks were randomly selected from fifteen randomly chosen nest boxes at ten days of age. Once collected, tissues were dissected out within the shortest period possible (i.e., 3 days). Measurements of body morphometrics were completed, including weight (g), wing chord length (mm), and ninth primary length (mm) before euthanasia. Following decapitation, breast tissue (0.53 – 1.39 g) was collected from the chicks for stable isotope analysis (n = 15). Livers were dissected from chicks and divided into aliquots for the analysis of PFAA concentrations (n = 15) and FA signatures (n = 12). The entire GI tract, from esophagus to cloacal vent, was removed and collected for PFAAs analysis (n = 15), and the remaining carcass was kept for FA analysis (n = 15). All tissues were briefly flash frozen in liquid nitrogen and stored at -80°C until analysis. Samples were shipped frozen to SGS AXYS Analytical (Sidney, British Columbia, Canada) for PFAS analysis (livers, GI tracts), to Ján Veizer Stable Isotope Laboratory (formerly known as G.G. Hatch Laboratory, Ottawa University) (Ottawa, Ontario Canada) for stable isotope preparation and analysis (breast muscle), to McGill University (Sainte-Anne-de-Bellevue, Quebec, Canada) for FA signature analysis (remaining liver aliquot, whole carcasses).

Macroinvertebrate Collection

Aquatic macroinvertebrates were collected from Red Hill Creek, Hamilton, Ontario, approximately 250 m directly downstream from the tree swallow breeding colony, using Hester-Dendy round 14-plate samplers (Dynamic Aqua) with adapted methods[1,2,3]. Traps were placed in pairs at three different locations, approximately 50 meters apart, between 250 m and 450 m downstream of the tree swallow colony (Fig. 1) and deployed for 4 weeks overlapping tree swallow nestling development. Traps were secured to the downstream side of cinder blocks to allow for water flow. Cinder blocks were placed on top of the sediment in the stream, approximately 2 m deep, and secured with rope to a nearby tree. Upon collection, traps were rinsed with creek water and all macroinvertebrate samples were pooled. The creek water rinsate

(n = 2) was collected for QA/QC PFAS analysis. Aquatic invertebrate samples were then weighed and divided into separate aliquots for analysis of PFAS (4.5 g/sample) (n = 5) and FAs (2.5 g/sample) (n = 5).

Terrestrial macroinvertebrates were collected within the colony and in the area surrounding the colony (~ 8000 m^2) where tree swallows were observed feeding (Fig. 1). Sampling occurred daily for two weeks in May and June, parallel with the development of the sampled tree swallow chicks to accurately reflect diet. Sweep nets were used to collect macroinvertebrates by sweeping through the long grasses in the sampling area, and sweeping surveys were conducted by one individual, once a day for 0.5-1 hr. Samples were frozen at -20 ^oC for 24 hours, then sorted to remove all non-target species and vegetation, and pooled. Samples were weighed and divided into aliquots for PFAS analysis (4.5 g/sample) (n = 5), and fatty acids (2.5 g/sample) (n = 5). Aquatic and terrestrial invertebrate samples for perfluorinated analysis were stored at -20 ^oC, and for FAs analysis were stored at -80 ^oC until analysis.

Air, Water and Sediment Collection

Five sorbent-impregnated polyurethane foam air samplers (SIP-PFAS) in double-dome housing were deployed within the tree swallow colony to passively sample air contaminants for a 50-day period overlapping tree swallow breeding and nestling development. Four SIP-PAS were placed at the four directional corners of the colony and the fifth at the center of the colony (Fig. 1). SIP-PAS field blanks (n = 2) were collected during the deployment of the five SIP-PAS. Procedures for sample disk preparation and deployment closely followed procedures previously outlined[33]. SIP-PAS s were deployed at a height of ~ 1.5 m above ground level in the tree swallow colony. SIP-PAS s were removed from housing and placed in amber glass jars. Samples and blanks were stored at ambient room temperature until extraction and analysis.

Surface water samples were collected 5-d post tree swallow tissue collection in June 2021, directly from the wastewater effluent outflow, located approximately 250 m upstream from the colony (Fig. 1). High density polyethylene (HDPE) sample bottles were pre-rinsed with creek water, and samples (500 mL; n = 6) were collected from the top 8-10 cm of the water column. Samples were stored at -20 °C until analysis.

Surficial sediment samples were also collected 5-d post tree swallow tissue collection in June 2021. We sampled for surficial sediments within 3-5 m downstream of each Hester-Dendy

trap (Fig. 1) to avoid disturbance and contamination of the HD traps, but to ensure the sample was representative of epi-benthic macroinvertebrate habitat. Sample bottles were rinsed with Red Hill Creek water and emptied downstream of sampling location. Sediment from the top 6 cm of the creek bed was sampled. Two sediment samples were collected per sampling location. Sediment samples (5 g) (n = 6) were stored at -20 °C until analysis.

Stable Isotope and Fatty Acid analysis

Methods for stable isotope extraction and instrumental analysis were completed by the Ján Veizer Stable Isotope Laboratory (University of Ottawa), and have been previously described[4,5]. Briefly, lipid-extracted, homogenized, freeze-dried tree swallow breast muscle samples were weighed into tin capsules and loaded into an elemental analyzer. Samples were flash combusted and analyzed on a coupled isotope ratio mass spectrometer (IRMS; Delta Advantage by Thermo, Germany). An in-house quality control Double Crested Cormorant (2003) was analyzed with each set of samples, and their results were within an acceptable 3% relative percent difference.

For fatty acid analysis, lipids were extracted from tree swallow livers using procedures previously established and documented[6,7]. Liver samples (0.11 – 0.40 g) were homogenized in a solution of 2:1 chloroform:methanol containing 0.01 % butylated hydroxytoluene (BHT v/v/w). A solution of 0.9 % sodium chloride was added to the sample. Samples were vortexed and left overnight at 4 °C. Samples were then centrifuged, and the bottom chloroform layer was isolated, filtered through anhydrous sodium sulfate, and the solvent was evaporated with nitrogen to obtain the extracted lipids. Methylene chloride with 0.01 % BHT and Hilditch reagent was then added to the samples which were then heated at 100 °C for 1 hr to *trans*-esterify the FAs to their fatty acid methyl ester (FAME) analogues. FAMEs were reconstituted with hexane to a final concentration of 50 mg total FAME ml^{-1} hexane. Samples were analyzed on an Agilent 8860 gas chromatograph (GC: Agilent Technologies, Santa Clara, CA, USA) with a flame ionization detector. The inlet was used in split mode, except for samples < 1 mg FAME, which were instead diluted to 0.5 mg/ml FAME ml^{-1} hexane and run-in splitless mode. A total of 66 individual FAs[8] were quantified as mass percent (%) of total FAME.

Whole carcass (1.50 – 1.85 g) and macroinvertebrate (0.72 – 1.63 g) samples were analyzed similarly to liver samples, except for preparation. Samples were homogenized in a

solution of 2:1 chloroform:methanol containing 0.01 % butylated hydroxytoluene (BHT v/v/w) to prevent oxidation and left overnight at 4 °C. Samples were then filtered to remove homogenate and rinsed with chloroform before centrifuging. Duplicates were extracted and analyzed for all carcasses (*n* = 15), aquatic macroinvertebrates (*n* = 5), and terrestrial macroinvertebrates (*n* = 4). The relative percent difference for the duplicates averaged 14 % in carcasses, 11 % in aquatic macroinvertebrates, and 21 % in terrestrial macroinvertebrates. A standard reference material (Krill RM 8037) was run concurrently, and results were within 13 % of known values.

PFAS Analysis

Air SIP-PAS samples (n = 7) were analyzed by the Air Quality Processes and Research Section of ECCC (Toronto, Ontario). Methods for extraction and the instrumental analysis of samples has been previously described[8]. Briefly, accelerated solvent extraction was performed on each SIP-PAS disk to collect two fractions of extracts. SIP-PAS s were spiked with internal standards (25 ng for volatile n-PFAS, 0.5 ng for ionizable PFAS; Table S2) prior to extraction. The first fraction of extracts, containing ionizable PFAS was extracted with acetonitrile (3 cycles), and the second fraction, containing volatile n-PFAS, was extracted using petroleum ether/acetone (85/15, v/v; 3 cycles). Ionizable PFAS were reduced to 0.5 mL using TurboVap (Biotage, Charlotte, NC) and nitrogen blowdown, followed by carbon column clean up with ENVI-Carb (100 mg; 100-400 mesh), volatile n-PFAS were reduced to 0.5 mL using rotary evaporation and nitrogen blowdown. Ionizable PFAS were washed with acetonitrile and reduced to 0.5 mL. Samples were spiked with injection standards prior to instrument analysis.

Analysis and detection of volatile n-PFAS was performed using ultra performance liquid chromatography coupled with tandem mass spectrometer (UPLC-MS/MS; Waters'). Ionizable PFAS were analyzed using gas chromatography and single quadrupole mass spectrometry (GC/MS; Agilent). Mass of target analytes in the samples were converted to corresponding air concentrations using the effective air volume (Veq.) sampled by SIPs. Veq was determined using the GAPS template using the average temperature during the deployment period and octanol-air partition coefficients for each target chemical. Standard recoveries for ionizable PFAS were within 55 – 90 %, and for volatile n-PFAS 70 – 135 %.

Extraction and instrumental analysis of water (*n* = 6), sediment (*n* = 6), aquatic macroinvertebrates (*n* = 5), terrestrial macroinvertebrates (*n* = 5), tree swallow GI tracts (*n* = 15), and livers (*n* = 15), was completed by SGS AXYS Analytical (Sidney, British Columbia, Canada) using EPA Method 1633 (SGS AXYS MLA-110) and has previously been described[36]. Extraction procedures differed slightly based on media.

Water samples were spiked with surrogate standards (Table S2) and extraction by solid phase extraction (SPE) using weak anion exchange cartridges. Sample extracts are then treated with carbon powder and spiked with recovery standards before analysis.

Sediment, macroinvertebrate, and tree swallow tissue samples were spiked with isotopically labelled surrogate standards, followed by extraction with methanolic ammonium hydroxide solution to collect supernatants. Supernatants are combined and samples are treated with ultra-pure carbon powder and evaporated. Samples are cleaned by SPE using cartridges containing a weak anion exchange sorbent. Samples were then spiked with recovery standards before analysis.

Identification and quantification of 46 PFAS (see Table S1 for full chemical names) was performed on an ultrahigh performance liquid chromatography (UPLC-MS/MS) reversed phase C18 column using a solvent gradient, using electrospray ionization in negative ionization mode and in multiple reaction monitoring mode. Surrogate standard recoveries ranged from 48-166 %. Minimum detection limits for all PFAS analyzed in all compartments is presented in Table S3.

Results

PFAS Detection Frequencies

Detection rates of PFAS congeners varied greatly between the environmental compartments sampled (Table 1). Detection frequencies in air samples and surface water samples of the C_4-C_{10} PFCAs (PFBA, PFPeA, PFHxA, PFHpA, PFOA, PFNA, and PFDA) were 100 %, but ranged from not detected to 100 % detected in the sediment, invertebrate and tree swallow samples. The latter samples showed high detections for the longer-chain C_{12}-C_{14} PFCAs (PFDoA, PFTrDA, and PFTeDA), but these compounds were not detected in air or surface water. All seven compartments had PFOS detection rates of 100 %. Sediment, aquatic invertebrates, and tree swallow GI tracts showed 100 % detection of PFDS, but all other long-chain C_9-C_{12} PFSAs (PFNS, PFDS, and PFDoS) were generally not detected in any samples.

Sediment, aquatic invertebrates, and both tree swallow tissues had detectable concentrations of 5:3 FTCA and, in both tree swallow tissues, 7:3 FTCA was also consistently detected. The perfluorooctane sulfonamidoacetic acids, N-MeFOSAA and N-EtFOSAA, were 100 % detected for all compartments, except that they were not analyzed in air samples. Air samples had detectable concentrations of the perfluorooctane sulfonamide ethanols, N-MeFOSE, and N-EtFOSE, which were much more variable in all other environmental compartments. For the terrestrial invertebrate samples, the precursor 6:2 FTS was the only PFAS congener detected. The per- and polyfluoroether carboxylates and ether sulfonates were not detected in any compartments, except for limited detection of 9Cl-PF3ONS in aquatic invertebrates.

Table S1- Subclass, number of perfluorinated carbons, and acronym of 46 target per- and polyfluoroalkyl substances analyzed in environmental compartments (air, surface water, sediment, aquatic and terrestrial invertebrates, and tree swallow gastrointestinal tracts and livers). Compounds analyzed in air denoted by [a], and compounds measured in surface water, sediment, aquatic and terrestrial invertebrates, and tree swallow gastrointestinal tracts and livers are denoted by [b].

Subclass	Target Compound	Number of Perfluorinated Carbons	Acronym
Perfluorocarboxylic acids	Perfluoro-n-butanoic acid	C_4	PFBA
	Perfluoro-n-pentanoic acid	C_5	PFPeA
	Perfluoro-n-hexanoic acid	C_6	PFHxA
	Perfluoro-n-heptanoic acid	C_7	PFHpA
	Perfluoro-n-octanoic acid	C_8	PFOA
	Perfluoro-n-nonanoic acid	C_9	PFNA
	Perfluoro-n-decanoic acid	C_{10}	PFDA
	Perfluoro-n-undecanoic acid	C_{11}	PFUnA
	Perfluoro-n-dodecanoic acid	C_{12}	PFDoA
	Perfluoro-n-tridecanoic acid	C_{13}	PFTrDA
	Perfluoro-n-tetradecanoic acid	C_{14}	PFTeDA
	Perfluoro-n-hexadecanoic acid	C_{16}	PFHxDA
	Perfluoro-n-octadecanoic acid	C_{18}	PFODA
Perfluorosulfonic acids	Perfluoro-1-n-butane sulfonic acid	C_4	PFBS
	Perfluoro-1-pentane sulfonic acid	C_5	PFPeS
	Perlfuoro-1-hexane sulfonic acid	C_6	PFHxS
	Perfluoro-1-heptane sulfonic acid	C_7	PFHpS
	Perfluoro-1-octane sulfonic acid	C_8	PFOS
	Perfluoro-n-onane sulfonic acid	C_9	PFNS
	Perfluoro-n-decane sulfonic acid	C_{10}	PFDS
	Perfluoro-n-dodecane sulfonic acid	C_{12}	PFDoS
Fluorotelomer sulfonates	1H, 1H, 2H, 2H-perfluorohexane sulfonic acid	C_4	4:2 FTS
	1H, 1H, 2H, 2H-perfluorooctane sulfonic acid	C_6	6:2 FTS
	1H, 1H, 2H, 2H-perfluorodecane sulfonic acid	C_8	8:2 FTS
	1H, 1H, 2H, 2H-perfluorododecane sulfonic acid	C_{10}	10:2 FTS
Fluorotelomer alcohols	1H, 1H, 2H, 2H-perfluoro-1-octanol	C_6	6:2 FTOH
	1H, 1H, 2H, 2H-perfluoro-1-decanol	C_8	8:2 FTOH
	1H, 1H, 2H, 2H-perfluoro-1-dodecanol	C_{10}	10:2 FTOH
Fluorotelomer carboxylates	2H, 2H, 3H, 3H-perfluorohexanoic acid	C_3	3:3 FTCA
	2H, 2H, 3H, 3H-perfluorooctanoic acid	C_5	5:3 FTCA
	2H, 2H, 3H, 3H-perfluorodecanoic acid	C_7	7:3 FTCA
Perfluorooctane sulfonamides	Perfluoro-1-octane sulfonamide	C_8	PFOSA
	N-Methyl-perfluoro-octane sulfonamide	C_8	N-MeFOSA
	N-Ethyl-perfluoro-octane sulfonamide	C_8	N-EtFOSA
Perfluorooctane sulfonamidoacetic acids	N-Methylperfluoro-1-octanesulfonamideoacetic acid	C_8	N-MeFOSAA
	N-Ethylperfluoro-1-octanesulfonamidoacetic acid	C_8	N-EtFOSAA
Perfluorooctane sulfonamide ethanols	N-Methylperfluoro-1-octanesulfonamidoacetic acid	C_8	N-MeFOSE
	N-Ethyl-perfluoro-octane sulfonamidoethanol	C_8	N-EtFOSE
Per- and polyfluoroether carboxylates	Hexafluoropropylene ide-dimer acid	C_5	HFPO-DA
	Decafluoro-3H-4,8-dioxanonoate	C_5	ADONA
	Perfluoro-3,6-dioxanonoate	C_4	NFDHA
	Perfluoro-3-methoxypropanoate	C_3	PFMPA
	Perfluoro-4-methoxybutanoate	C_4	PFMBA
Ether sulfonates	9-chlorohexadecafluoro-3-oxanonane-1-sulfonic acid	C_8	9CI-PF3ONS
	11-chloroeicosafluoro-3-oxaundecane-1-sulfonic acid	C_{10}	11CI-PF3OUdS
	Perfluoro (2-ethoxyethane) sulfonic acid	C_4	PFEESA

Table S2 - Surrogate standards for perfluoroalkyl substance (PFAS) extraction and analysis in environmental samples collected from an aquatic-terrestrial food web in the Laurentian Great Lakes Basin in southwestern Ontario, Canada. See Table S1 for full PFAS names.

Target Compound	Surrogate Standard
Air	
PFBA	13C4-PFBA
PFHxA	13C2-PFHxA
PFHxS	18O2-PFHxS
PFNA	13C5-PFNA
PFOA	13C4-PFOA
PFOS	13C4-PFOS
PFDA	13C2-PFDA
PFUDA	13C2-PFUDA
PFDoA	13C2-PFDoA
6:2 FTOH	M6:2 FTOH
8:2 FTOH	M8:2 FTOH
10:2 FTOH	M10:2 FTOH
EtFOSA	D5-N-EtFOSA
MeFOSA	D3-N-MeFOSA
MeFOSE	D7-N-MeFOSE
EtFOSE	D9-N-EtFOSE
Surface Water, Sediment, Invertebrates, and Tree Swallow Tissues	
PFBA	13C4-PFBA
PFPeA	13C5-PFPeA
PFHxA	13C5-PFHxA
PFHpA	13C4-PFHpa
PFOA	13C8-PFOA
PFNA	13C9-PFNA
PFDA	13C6-PFDA
PFUnA	13C7-PFUnA
PFDoA	13C2-PFDoA
PFTeDA	13C2-PFTeDA
PFBS	13C3-PFBS
PFHxS	13C3-PFHxS
PFOS	13C8-PFOS
4:2 FTS	13C2-4:2 FTS
6:2 FTS	13C2-6:2 FTS
8:2 FTS	13C2-8:2 FTS
PFOSA	13C8-PFOSA
MeFOSA	D3-N-MeFOSA
EtFOSA	D5-N-EtFOSA
MeFOSA	D3-N-MeFOSA
EtFOSA	D5-N-EtFOSA
MeFOSAA	D3-N-MeFOSAA
EtFOSAA	D5-N-EtFOSAA
MeFOSE	D7-N-MeFOSE
EtFOSE	D9-N-EtFOSE
HFPO-DA	13C3-HFPO-DA

Table S3 - Detection frequencies (%) of perfluorocarboxylic acids (PFCAs), perfluorosulfonic acids (PFSAs), and) precursors sampled in air, surface water, sediment, aquatic invertebrates, terrestrial invertebrates, and tree swallow nestling GI tracts and livers near a wastewater treatment plant site in southwestern Ontario, Canada (See Figure 1). Congeners not analyzed within a given compartment are denoted with an X. See Table S2 for full PFAS chemical names.

Subclass	Acronym	Detection frequency (%)						
		Air<br>*n* = 5	SW[a]<br>*n* = 6	S[a]<br>*n* = 6	AI[a]<br>*n* = 5	TI[a]<br>*n* = 5	GI[a,b]<br>*n* = 15	Livers[b]<br>*n* = 15
Perfluorocarboxylic acids (PFCAs)	PFBA	100	100	0	0	0	0	27
	PFPeA	0	100	0	60	0	7	0
	PFHxA	100	100	100	100	0	0	0
	PFHpA	100	100	0	0	0	0	0
	PFOA	100	100	67	60	0	40	0
	PFNA	100	100	0	0	0	93	100
	PFDA	100	100	100	60	0	100	100
	PFUnA	0	0	33	0	0	100	100
	PFDoA	0	0	100	100	0	100	7
	PFTrDA	100	0	83	100	0	100	7
	PFTeDA	0	0	100	100	0	100	0
	PFHxDA	0	X	X	X	X	X	X
	PFODA	0	X	X	X	X	X	X
Perfluorosulfonic acids (PFSAs)	PFBS	100	100	0	0	0	0	0
	PFPeS	X	67	0	0	0	0	0
	PFHxS	100	100	0	0	0	0	0
	PFHpS	X	0	0	0	0	13	0
	PFOS	100	100	100	100	100	100	100
	PFNS	X	0	33	0	0	0	0
	PFDS	0	0	100	100	0	100	7
	PFDoS	X	0	0	0	0	0	0
Fluorotelomer sulfonates	4:2 FTS	X	0	0	0	0	0	0
	6:2 FTS	80	100	0	20	80	0	0
	8:2 FTS	60	0	0	0	0	0	0
	10:2 FTS	60	X	X	X	X	X	X
Fluorotelomer alcohols	6:2 FTOH	100	X	X	X	X	X	X
	8:2 FTOH	100	X	X	X	X	X	X
	10:2 FTOH	100	X	X	X	X	X	X
Fluorotelomer carboxylates	3:3 FTCA	X	0	0	0	0	0	0
	5:3 FTCA	X	0	33	100	0	80	40
	7:3 FTCA	X	0	0	0	0	87	80
Perfluorooctane sulfonamides	PFOSA	X	0	100	100	0	60	0
	N-MeFOSA	100	0	0	0	0	80	0
	N-EtFOSA	100	0	0	0	0	67	0
Perfluorooctane sulfonamidoacetic acids	N-MeFOSAA	X	100	100	100	0	100	67
	N-EtFOSAA	X	100	100	100	0	100	67
Perfluorooctane sulfonamide ethanols	N-MeFOSE	100	0	0	0	0	0	0
	N-EtFOSE	100	0	0	20	0	87	13
Per- and polyfluoroether carboxylates	HFPO-DA	X	0	0	0	0	0	0
	ADONA	X	0	0	0	0	0	0
	NFDHA	X	0	0	0	0	0	0
	PFMPA	X	0	0	0	0	0	0
	PFMBA	X	0	0	0	0	0	0
Ether sulfonates	9CI-PF3ONS	X	0	0	40	0	0	0
	11CI-PF3OUdS	X	0	0	0	0	0	0
	PFEESA	X	0	0	0	0	0	0

[a] SW = surface water, S = sediment, AI = aquatic invertebrates, TI = terrestrial invertebrates, GI = gastrointestinal tracts

[b] Tree swallow nestling tissues

Table S4- Instrument limit of detection (IDL) in air samples and minimum limit of detection (MLOD) in surface water, sediment, aquatic invertebrates, terrestrial invertebrates, and tree swallow nestling gastrointestinal tracts (GI tracts) and livers for 46 per- and polyfluoroalkyl substances. Congeners not analyzed within a given compartment are denoted with an X See Table S1 for full PFAS names.

Subclass	Congener	Air (pg/m3)	SW[a] (ng/L)	S[a] (ng/g dw)	AI[a] (ng/g ww)	TI[a] (ng/g ww)	GI[a,b] (ng/g ww)	Liver[b] (ng/g ww)
Perfluorocarboxylic acids	PFBA	5.1	1.6	1.2	0.4	1.1	0.4	2.3
	PFPeA	0.14	0.8	0.6	0.2	0.3	0.2	1.1
	PFHxA	0.01	0.4	0.3	0.1	0.1	0.1	0.6
	PFHpA	0.00	0.4	0.3	0.1	0.1	0.1	0.6
	PFOA	0.01	0.4	0.3	0.1	0.1	0.1	0.6
	PFNA	0.02	0.4	0.3	0.1	0.1	0.1	0.6
	PFDA	1.82	0.4	0.3	0.1	0.1	0.1	0.6
	PFUnA	0.15	0.4	0.3	0.1	0.1	0.1	0.6
	PFDoA	0.20	0.4	0.3	0.1	0.1	0.1	0.6
	PFTrDA	0.19	0.4	0.3	0.1	0.1	0.1	0.6
	PFTeDA	0.21	0.4	0.3	0.1	0.1	0.1	0.6
Perfluorosulfonic acids	PFBS	0.05	0.4	0.3	0.1	0.1	0.1	0.6
	PFPeS	X	0.4	0.3	0.1	0.1	0.1	0.6
	PFHxS	0.05	0.4	0.3	0.1	0.1	0.1	0.6
	PFHpS	X	0.4	0.3	0.1	0.1	0.1	0.6
	PFOS	0.03	0.4	0.3	0.1	0.1	0.1	0.6
	PFNS	X	0.4	0.3	0.1	0.1	0.1	0.6
	PFDS	X	0.4	0.3	0.1	0.1	0.1	0.6
	PFDoS	X	0.4	0.3	0.1	0.1	0.1	0.6
Fluorotelomer sulfonates	4:2 FTS	X	1.6	1.2	0.4	0.4	0.4	2.3
	6:2 FTS	X	1.5	1.0	0.3	0.4	0.4	2.0
	8:2 FTS	X	1.6	1.2	0.4	0.4	0.4	2.3
Fluorotelomer alcohols	6:2 FTOH	0.04	X	X	X	X	X	X
	8:2 FTOH	0.04	X	X	X	X	X	X
	10:2 FTOH	0.03	X	X	X	X	X	X
Fluorotelomer carboxylates	3:3 FTCA	X	1.6	1.2	0.4	0.4	0.4	2.3
	5:3 FTCA	X	10.3	7.2	2.4	2.5	2.7	14.2
	7:3 FTCA	X	10.3	7.2	N/A	2.5	2.7	14.2
Perfluorooctane sulfonamides	PFOSA	X	0.4	0.3	0.1	0.1	0.1	0.6
	N-MeFOSA	0.02	0.5	0.3	0.1	0.1	0.2	0.7
	N-EtFOSA	0.02	1.0	0.7	0.2	0.2	0.3	1.4
Perfluorooctane sulfonamidoacetic acids	N-MeFOSAA	X	0.4	0.3	0.1	0.1	0.1	0.6
	N-EtFOSAA	X	0.4	0.3	0.1	0.1	0.1	0.6
Perfluorooctane sulfonamide ethanols	N-MeFOSE	0.03	4.1	2.9	1.0	1.0	1.3	5.7
	N-EtFOSE	0.05	3.1	2.2	0.7	0.7	0.8	4.2
Per- and polyfluoroether carboxylates	HFPO-DA	X	1.6	1.1	0.4	0.4	0.4	2.2
	ADONA	X	1.6	1.2	0.4	0.4	0.4	2.3
	NFDHA	X	0.8	0.6	0.3	0.2	0.2	1.1
	PFMPA	X	0.8	0.6	0.2	0.2	0.2	1.1
	PFMBA	X	0.4	0.3	0.1	0.1	0.1	0.6
Ether Sulfonates	9Cl-PF3ONS	X	1.6	1.2	0.4	0.4	0.4	2.3
	11Cl-PF3OUdS	X	1.6	1.2	0.4	0.4	0.4	2.3
	PFEESA	X	0.4	0.3	0.1	0.1	0.1	0.6

[a] SW = surface water, S = sediment, AI = aquatic invertebrates, TI = terrestrial invertebrates, GI = gastrointestinal tracts

[b] Tree swallow nestling tissues

Table S5 - Perfluoroalkyl substances (PFAS) that were detected in ≥ 60 % of samples and therefore included in subsequent statistical analyses. Checks indicate congeners ≥ 60 % detection frequency in the respective compartment, NA indicates ≤ 60 % detection frequency, or the congener wasn't analyzed.

Subclass	Congener	Air	SW[a]	S[a]	AI[a]	TI[a]	GI[a,b]	Liver[b]
Perfluorocarboxylic acids	PFBA	✓	✓	NA	NA	NA	NA	NA
	PFPeA	NA	✓	NA	✓	NA	NA	NA
	PFHxA	✓	✓	✓	✓	NA	NA	NA
	PFHpA	✓	✓	NA	NA	NA	NA	NA
	PFOA	✓	✓	✓	✓	NA	NA	NA
	PFNA	✓	✓	NA	NA	NA	✓	✓
	PFDA	✓	✓	✓	✓	NA	✓	✓
	PFUnA	NA	NA	NA	NA	NA	✓	✓
	PFDoA	NA	NA	✓	✓	NA	✓	NA
	PFTrDA	✓	NA	✓	✓	NA	✓	NA
	PFTeDA	NA	NA	✓	✓	NA	✓	NA
Perfluorosulfonic acids	PFBS	✓	✓	NA	NA	NA	NA	NA
	PFPeS	NA	✓	NA	NA	NA	NA	NA
	PFHxS	✓	✓	NA	NA	NA	NA	NA
	PFHpS	NA	NA	NA	NA	NA	NA	NA
	PFOS	✓	✓	✓	✓	✓	✓	✓
	PFDS	NA	NA	✓	✓	NA	✓	NA
Fluorotelomer sulfonates	6:2 FTS	✓	✓	NA	NA	✓	NA	NA
	8:2 FTS	✓	NA	NA	NA	NA	NA	NA
	10:2 FTS	✓	NA	NA	NA	NA	NA	NA
Fluorotelomer alcohols	6:2 FTOH	✓	NA	NA	NA	NA	NA	NA
	8:2 FTOH	✓	NA	NA	NA	NA	NA	NA
	10:2 FTOH	✓	NA	NA	NA	NA	NA	NA
Fluorotelomer carboxylates	5:3 FTCA	NA	NA	NA	✓	NA	✓	NA
	7:3 FTCA	NA	NA	NA	NA	NA	✓	✓
Perfluorooctane sulfonamides	PFOSA	NA	NA	✓	✓	NA	✓	NA
	N-MeFOSA	✓	NA	NA	NA	NA	✓	NA
	N-EtFOSA	✓	NA	NA	NA	NA	✓	NA
Perfluorooctane sulfonamidoacetic acids	N-MeFOSAA	NA	✓	✓	✓	NA	✓	✓
	N-EtFOSAA	NA	✓	✓	✓	NA	✓	✓
Perfluorooctane sulfonamide ethanols	N-MeFOSE	✓	NA	NA	NA	NA	NA	NA
	N-EtFOSE	✓	NA	NA	NA	NA	✓	NA

[a] SW = surface water, S = sediment, AI = aquatic invertebrates, TI = terrestrial invertebrates, GI = gastrointestinal tracts

[b] Tree swallow nestling tissues

Table S6 - A comparison of five dietary fatty acids from aquatic invertebrates (AI), terrestrial invertebrates (TI), and tissues of tree swallow nestlings (i.e., livers, carcasses). Means ± SEMs are presented, along with the results of Pearson/Spearman's correlations of fatty acids with the principal components generated by the principal component analysis (PCA) of FA proportions of these biota. See Fig. 2 for PCA biplot.

Fatty Acid	Aquatic Invertebrates	Terrestrial Invertebrates	Tree Swallow Nestling		Overall Model	*Post-hoc* Results	PC1		PC2	
			Liver	Carcass						
	(*n* = 5)	(*n* = 5)	(*n* = 11)	(*n* = 13)			rho	*p*	rho	*p*
18:2n6	6.33 ± 0.19	12.08 ± 0.32	8.41 ± 0.28	10.36 ± 0.08	$F_{4,29} = 65.73$, $p < 0.001$	TI > Carcasses > Livers > AI	**-0.08**	**<0.001**	-0.1	0.58
18:3n3	5.56 ± 0.34	10.17 ± 0.68	0.93 ± 0.05	3.04 ± 0.13	$F_{4,29} = 297.6$, $p < 0.001$	TI > AI > Carcasses > Livers	**-0.74**	**<0.001**	**0.53**	**0.002**
20:4n6	3.83 ± 0.17	0.58 ± 0.01	7.25 ± 0.64	1.59 ± 0.05	$F_{4,29} = 38.02$, $p < 0.001$	Livers > AI > Carcasses = TI	0.22	0.21	**0.82**	**<0.001**
20:5n3	7.4 ± 0.19	1.40 ± 0.06	3.16 ± 0.40	2.66 ± 0.10	$F_{4,29} = 38.88$, $p < 0.001$	AI > TI = Livers = Carcasses	**0.65**	**<0.001**	**0.35**	**0.04**
22:6n3	0.98 ± 0.02	0.08 ± 0.004	6.76 ± 0.46	0.87 ± 0.92	$F_{4,29} = 85.79$, $p < 0.001$	Livers > AI > Carcasses = TI	**0.92**	**<0.001**	-0.32	0.07

Table S7 - Comparisons of perfluoroalkyl substance (PFAS) concentrations among abiotic and biotic compartments of a tree swallow food web. PFAS concentrations of terrestrial insects were excluded from these statistical comparisons except for PFOS and 6:2 FTS, since the remaining PFAS congeners were undetected (Table S4). Surface water (water), sediment (S), aquatic invertebrates (AI), terrestrial invertebrates (TI), nestling gastrointestinal tracts (GIT), nestling livers. See Table S1 for full PFAS names.

Subclass	Congener	Overall Model	*Post-hoc* Results
Short-chain PFCA	PFPeA	$F_{1,9}$ = 438.3 p <0.001	Water > AI
	PFHxA	$F_{3,18}$ = 65.31 p <0.001	S > AI > Air = Water
Long-chain PFCA	PFOA	$F_{3,18}$ = 191.9 p <0.001	S > AI > Air = Water
	PFNA	$F_{3,37}$ = 7.92 p <0.001	Liver > GIT > Water = Air
	PFDA	$F_{5,44}$ = 73.74 p <0.001	Liver > GIT > S > AI > Air > Water
	PFUnA	$F_{1,23}$ = 118.1 p <0.001	Liver > GIT
	PFDoA	$F_{3,23}$ = 14.15 p <0.001	S = AI > GIT
	PFTrDA	$F_{4,28}$ = 21.93 p <0.001	S = AI = GIT > Air
	PFTeDA	$F_{2,23}$ = 8.78 p = 0.001	S > GIT = AI
Long-chain PFSA	PFOS	$F_{6,50}$ = 112.7 p <0.001	Liver > GIT > TI = S = AI > Air > Water
	PFDS	$F_{3,23}$ = 34.15 p <0.001	S > GIT = AI
Fluorotelomer sulfonamides	6:2 FTS	$F_{3,13}$ = 591.3 p <0.001	TI > Air > Water
Fluorotelomer carboxylates	5:3 FTCA	$F_{1,17,}$ = 10.08 p = 0.16	AI = GIT
	7:3 FTCA	$F_{1,28}$ = 40.74 p <0.001	Liver > GIT
Perfluorooctane sulfonamides	PFOSA	$F_{2,23}$ = 17.46 p <0.001	S = AI > GIT
	N-MeFOSA	$F_{1,18}$ = 17.09 p <0.001	GIT > Air
	N-EtFOSA	$F_{1,18}$ = 24.84 p <0.001	GIT > Air
Perfluorooctane sulfonamidoacetic acids	N-MeFOSAA	$F_{4,42}$ = 10.38 p <0.001	S > Liver = AI = GIT > Water
	N-EtFOSAA	$F_{4,42}$ = 15.75 p <0.001	S > Liver = AI > GIT > Water
Perfluorooctane sulfonamide ethanols	N-EtFOSE	$F_{1,18}$ = 14.52 p <0.001	GIT > Air

Table S8 – Standard deviations of calculated bioaccumulation factors (BAFs) and biomagnification factors (BMFs) for per- and polyfluoroalkyl substances (PFAS) in abiotic and biotic compartments of a tree swallow food web in southwestern Ontario in 2021. Nestling samples involved their gastrointestinal tract and livers.

		Short-Chain PFCAs		Long-Chain PFCAs					Long-Chain PFSAs		Precursors							
		PFPeA	PFHxA	PFOA	PFNA	PFDA	PFDoA	PFTrDA	PFOS	PFDS	6:2FTS	5:3 FTCA	PFOSA	N-MeFOSA	N-EtFOSA	N-MeFOSAA	N-EtFOSAA	N-EtFOSE
BAF	AI/SW[a]	0.97	4.99	2.26	-	17.2	-	-	31.2	-	-	-	-	-	-	19.5	0.62	-
	AI/Sediment[a]	-	0.20	0.28	-	-	0.36	0.48	0.52	0.11	-	-	0.63	-	-	0.30	762	-
	GI/SW[a]	-	-	-	102	226	-	-	395	-	-	-	-	-	-	12.0	139	-
	Liver/SW[a]	-	-	-	279	-	-	-	1148	-	-	-	-	-	-	26.6	276	-
	TI/Air[a]	-	-	-	-	-	-	-	20.7	-	96.0	-	-	-	-	-	-	-
	GI/Air[a]	-	-	-	229	138	-	21.8	96.3	-	-	-	-	240	115	-	-	176
	Liver/Air	-	-	-	818	419	-	-	291	-	-	-	-	-	-	-	-	-
BMF	GI/AI[a]	-	-	-	-	2.22	0.15	0.13	1.68	0.27	-	0.58	0.10	-	-	0.31	0.30	-
	Liver/AI[a]	-	-	-	-	5.88	-	-	4.90	-	-	-	-	-	-	0.71	0.65	-
	GI/TI[a]	-	-	-	-	-	-	-	1.43	-	-	-	-	-	-	-	-	-
	Liver/TI[a]	-	-	-	-	-	-	-	4.24	-	-	-	-	-	-	-	-	-

[a] SW = surface water, S = sediment, AI = aquatic invertebrates, TI = terrestrial invertebrates, GI = gastrointestinal tracts

Table S9 – Correlations of the first two dimensions of a multiple factor analysis (MFA) with the concentrations of individual PFAS congeners and precursors measured in abiotic (i.e., air, surface water, sediment) and biotic (i.e., aquatic and terrestrial macroinvertebrates, tree swallow nestling gastrointestinal tracts and livers) samples collected from an aquatic-terrestrial food web in southwestern Ontario. See Fig. 4 for the MFA variables and statistical results.

Congener	Dimension 1		Dimension 2	
	rho	*p-value*	rho	*p-value*
PFBA	**-0.34**	**0.009**	0.22	0.10
PFPeA	0.24	0.07	-0.03	0.80
PFHxA	**0.77**	**<0.001**	0.19	0.16
PFHpA	-0.10	0.47	**-0.85**	**<0.001**
PFOA	**0.80**	**<0.001**	0.25	0.06
PFNA	**-0.70**	**<0.001**	**0.62**	**<0.001**
PFDA	**-0.61**	**<0.001**	**0.68**	**<0.001**
PFUnA	**-0.68**	**<0.001**	**0.67**	**<0.001**
PFDoA	**0.87**	**<0.001**	**0.35**	**0.009**
PFTrDA	**0.76**	**<0.001**	**0.39**	**0.003**
PFTeDA	**0.88**	**<0.001**	**0.33**	**0.01**
PFBS	-0.11	0.43	**-0.89**	**<0.001**
PFHxS	-0.10	0.47	**-0.85**	**<0.001**
PFOS	**-0.67**	**<0.001**	**0.66**	**<0.001**
PFDS	**0.73**	**<0.001**	**0.36**	**<0.001**
6:2 FTS	0.16	0.23	-0.04	0.77
N-MeFOSA	0.13	0.32	0.25	0.06
N-EtFOSA	0.15	0.26	0.25	0.06
N-MeFOSE	-0.07	0.63	**-0.55**	**<0.001**
N-EtFOSE	-0.07	0.61	**0.29**	**0.03**

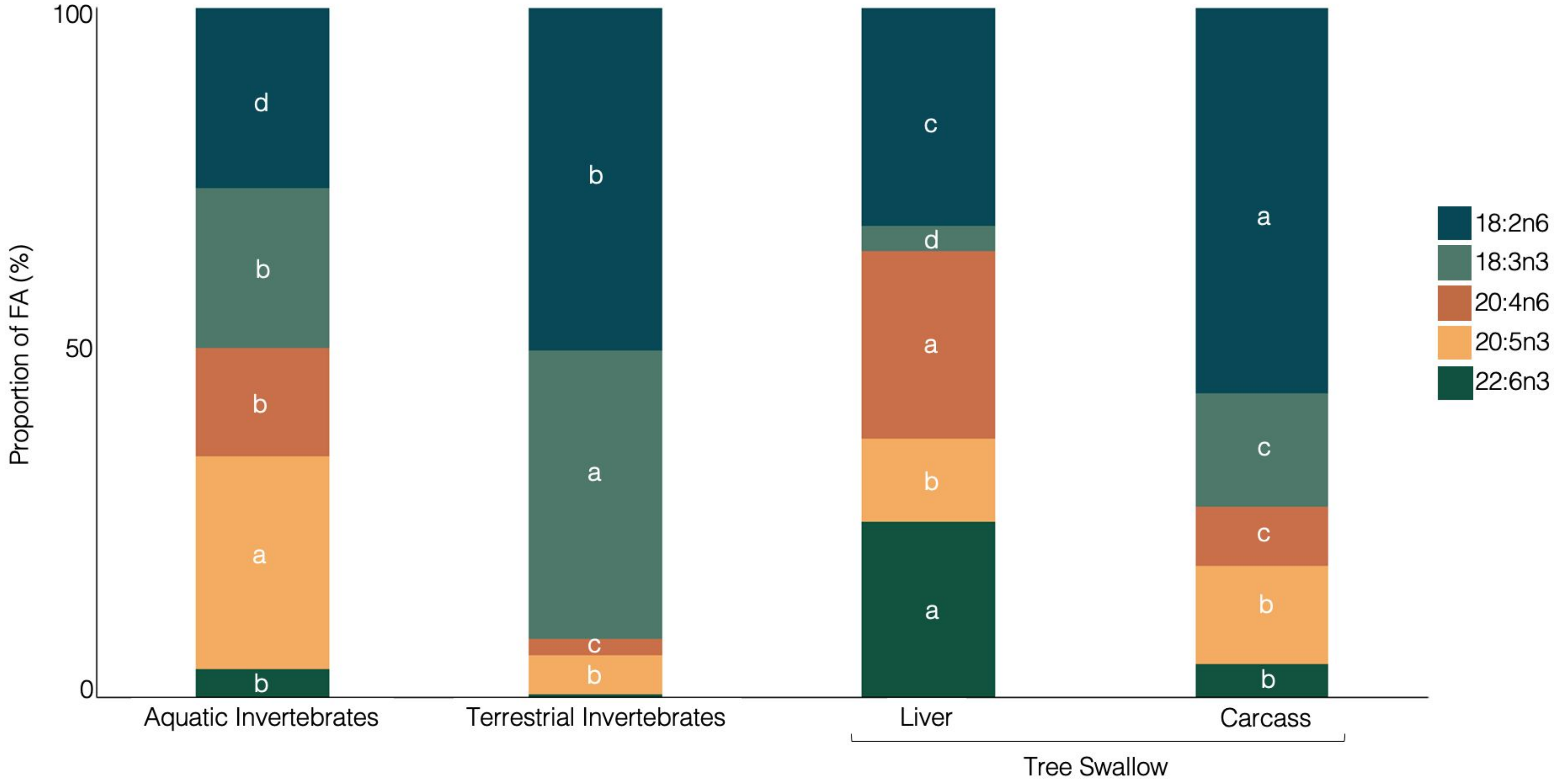

Fig. S1 - Profiles of five selected dietary fatty acid (FA) proportions (% of ∑FA proportions) measured in aquatic invertebrates and terrestrial invertebrates, and in tree swallow nestling livers and carcasses. Letters denote significant differences in proportions between sample types (all $p \leq 0.05$). Only the major dietary FAs, those with proportions greater than 0.1 % were examined, and we selected the top 5 major FAs in the tree swallow nestling carcass for statistical analysis.

Table S10 – Values of stable carbon ($\delta^{13}C$) and nitrogen ($\delta^{15}N$) isotopes for all tree swallow nestling breast muscle samples collected in 2021 from an aquatic-terrestrial ecosystem in southwestern Ontario.

Sample ID	Tissue	$\delta^{13}C$ (‰)	$\delta^{15}N$ (‰)
WBS-1	Breast Muscle	-23.9	12.5
WBS-3	Breast Muscle	-23.6	12.8
WBS-4	Breast Muscle	-23.5	12.3
WBS-7	Breast Muscle	-23	12.7
WBS-12	Breast Muscle	-24	13.1
WBS-13	Breast Muscle	-23	12.3
WBS-18	Breast Muscle	-22.5	12.7
WBS-24	Breast Muscle	-23.1	14
WBS-29	Breast Muscle	-23.6	12.3
WBS-32	Breast Muscle	-23.7	12.2
WBS-34	Breast Muscle	-22.7	13.6
WBS-37	Breast Muscle	-23.8	12.5
WBS-38	Breast Muscle	-22.7	12.9
WBS-41	Breast Muscle	-23.2	13.2
WBS-49	Breast Muscle	-23.4	12.1

Table S11 – Proportions of individual fatty acids relative to the sum of the 66 fatty acids quantified in aquatic invertebrate, terrestrial invertebrate, and tree swallow liver and carcass samples collected in 2021 from an aquatic-terrestrial food web in southwestern Ontario.

Sample ID	Compartment	8:0	10:0	12:0	13:0	iso14:0	14:0	14:1n9	14:1n7	14:1n5	iso15:0	anti15:0	15:0	15:1n8	15:1n6
HD01	Aquatic Invertebrates	0.0075	< MLOD	0.357	0.1975	0.5715	1.733	0.1855	0.119	0.1835	1.6815	0.576	0.6575	0.028	0.101
HD02	Aquatic Invertebrates	0.009	< MLOD	0.367	0.185	0.5385	1.7315	0.136	0.146	0.2265	1.436	0.485	0.5935	0.028	0.106
HD03	Aquatic Invertebrates	0.0085	< MLOD	0.3345	0.1685	0.502	1.6915	0.1415	0.11	0.1915	1.3595	0.4545	0.567	0.0265	0.091
HD04	Aquatic Invertebrates	0.009	0.02	0.338	0.1745	0.5245	1.7515	0.121	0.107	0.23	1.3965	0.4785	0.5725	0.028	0.103
HD05	Aquatic Invertebrates	0.006	< MLOD	0.344	0.1795	0.5245	1.707	0.128	0.122	0.207	1.395	0.4735	0.5805	0.024	0.086
TR01	Terrestrial Invertebrates	0.081	0.058	0.5445	0.011	0.037	2.0175	0.051	0.0665	0.664	0.0395	0.0515	0.1185	0.0045	0.0355
TR02	Terrestrial Invertebrates	0.111	0.1085	0.459	0.0105	0.0335	1.733	0.0565	0.0705	0.545	0.061	0.0405	0.1215	0.0045	0.038
TR03	Terrestrial Invertebrates	0.1525	0.069	0.5415	0.011	0.029	2.572	0.058	0.058	0.455	0.0665	0.0465	0.123	0.003	0.04
TR04	Terrestrial Invertebrates	0.085	< MLOD	0.434	0.014	0.033	2.394	0.054	0.077	0.442	0.08	0.046	0.128	0.005	0.036
TR05	Terrestrial Invertebrates	0.048	< MLOD	0.371	0.013	0.042	2.252	0.088	0.069	0.575	0.072	0.052	0.136	0.006	0.051
WBS-1L	Tree Swallow Liver	< MLOD	0.004	0.058	0.013	0.037	0.852	0.007	0.022	0.069	0.062	0.029	0.121	0.001	0.02
WBS-3L	Tree Swallow Liver	< MLOD	< MLOD	0.067	0.014	0.027	1.412	0.011	0.029	0.156	0.096	0.044	0.148	0.013	0.032
WBS-4L	Tree Swallow Liver	< MLOD	< MLOD	< MLOD	< MLOD	0.148	0.119	0.017	0.014	0.18	0.043	0.043	0.032	<MLOQ	0.032
WBS-7L	Tree Swallow Liver	0.007	< MLOD	0.047	0.009	0.033	1.269	0.011	0.022	0.047	0.1	0.04	0.127	0.006	0.026
WBS-12L	Tree Swallow Liver	< MLOD	< MLOD	0.057	0.014	0.017	1.208	0.002	0.024	0.075	0.096	0.042	0.141	<MLOQ	0.036
WBS-13L	Tree Swallow Liver	0.007	< MLOD	0.028	0.008	0.043	0.829	0.007	0.016	0.054	0.073	0.03	0.123	0.004	0.034
WBS-24L	Tree Swallow Liver	< MLOD	< MLOD	0.014	< MLOD	0.385	0.153	0.006	0.006	0.17	0.014	0.044	0.03	<MLOQ	0.016
WBS-29L	Tree Swallow Liver	0.007	< MLOD	0.044	0.012	0.032	1.558	0.009	0.024	0.128	0.136	0.053	0.164	0.006	0.028
WBS-32L	Tree Swallow Liver	< MLOD	< MLOD	0.029	< MLOD	0.027	0.571	0.015	0.016	0.035	0.051	0.02	0.127	<MLOQ	0.061
WBS-37L	Tree Swallow Liver	< MLOD	< MLOD	0.052	0.011	0.038	1.472	0.006	0.025	0.134	0.115	0.042	0.14	0.007	0.025
WBS-41L	Tree Swallow Liver	< MLOD	< MLOD	0.042	0.009	0.033	0.571	0.005	0.018	0.04	0.046	0.02	0.129	<MLOQ	0.021
WBS-49L	Tree Swallow Liver	0.015	< MLOD	0.073	0.021	0.019	0.589	0.006	0.033	0.074	0.045	0.023	0.149	<MLOQ	0.061
WBS-1C	Tree Swallow Carcass	0.0255	< MLOD	0.633	0.0465	0.077	3.904	0.0275	0.1645	0.322	0.2255	0.1445	0.3075	0.0095	0.054
WBS-3C	Tree Swallow Carcass	0.0455	< MLOD	0.783	0.0435	0.055	3.8315	0.034	0.1785	0.4935	0.203	0.1205	0.308	0.012	0.055
WBS-4C	Tree Swallow Carcass	0.027	< MLOD	0.8695	0.058	0.0735	4.413	0.033	0.1425	0.5435	0.2235	0.138	0.3345	0.012	0.0505
WBS-7C	Tree Swallow Carcass	0.0235	< MLOD	0.63	0.0485	0.062	4.0475	0.0345	0.187	0.4125	0.1945	0.117	0.2885	0.01	0.047

Sample ID	Compartment	8:0	10:0	12:0	13:0	iso14:0	14:0	14:1n9	14:1n7	14:1n5	iso15:0	anti15:0	15:0	15:1n8	15:1n6
WBS-12C	Tree Swallow Carcass	0.023	< MLOD	0.742	0.045	0.072	3.7185	0.0375	0.146	0.397	0.113	0.1405	0.317	0.011	0.054
WBS-13C	Tree Swallow Carcass	0.05	< MLOD	0.756	0.0595	0.1085	4.767	0.0355	0.1675	0.544	0.1385	0.168	0.3695	0.012	0.06
WBS-18C	Tree Swallow Carcass	0.0435	< MLOD	0.738	0.0515	0.0955	4.011	0.0295	0.155	0.424	0.2205	0.1405	0.3155	0.0115	0.0535
WBS-24C	Tree Swallow Carcass	0.0415	< MLOD	0.761	0.051	0.0845	3.7615	0.0395	0.1485	0.4975	0.2135	0.1305	0.3075	0.013	0.0505
WBS-29C	Tree Swallow Carcass	0.026	< MLOD	0.718	0.0525	0.097	4.185	0.034	0.1695	0.466	0.1255	0.1545	0.3285	0.0095	0.0545
WBS-32C	Tree Swallow Carcass	0.028	< MLOD	0.647	0.053	0.0895	3.691	0.035	0.169	0.476	0.2235	0.136	0.3175	0.011	0.058
WBS-34C	Tree Swallow Carcass	0.05	< MLOD	0.6185	0.0445	0.0885	3.5275	0.035	0.1585	0.2615	0.2305	0.1305	0.3255	0.0095	0.039
WBS-37C	Tree Swallow Carcass	0.032	< MLOD	0.8315	0.045	0.0775	4.291	0.0315	0.182	0.517	0.2085	0.126	0.296	0.01	0.05
WBS-38C	Tree Swallow Carcass	0.034	< MLOD	0.5705	0.0525	0.088	3.8245	0.033	0.1535	0.301	0.2505	0.15	0.35	0.01	0.0575
WBS-41C	Tree Swallow Carcass	0.034	< MLOD	0.729	0.051	0.089	4.1745	0.0335	0.171	0.358	0.121	0.1475	0.343	0.008	0.053
WBS-49C	Tree Swallow Carcass	0.059	< MLOD	0.6705	0.0505	0.0925	3.669	0.0415	0.1595	0.506	0.2175	0.1365	0.3195	0.008	0.0505

Sample ID	Compartment	iso16:0	16:0	16:1n11	16:1n9	16:1n7	7Me16:0	16:1n5	16:2n6	iso17:0	16:2n4	16:3n6	17:0	16:3n4	17:1
HD01	Aquatic Invertebrates	0.021	17.713	0.555	0.5545	12.8705	0.961	1.0625	0.5015	0.2925	0.413	0.4795	0.6145	0.6675	1.0685
HD02	Aquatic Invertebrates	0.026	17.266	0.4085	0.735	12.239	0.8295	0.4925	0.459	0.3665	0.4525	0.6205	0.5685	0.8255	1.3865
HD03	Aquatic Invertebrates	0.0245	16.5675	0.4955	0.6245	11.9075	0.8115	0.942	0.4235	0.3605	0.4315	0.609	0.561	0.8085	1.3025
HD04	Aquatic Invertebrates	0.0225	17.3955	0.3905	0.749	12.16	0.8105	0.025	0.4365	0.3765	0.4485	0.659	0.57	0.8545	1.3705
HD05	Aquatic Invertebrates	0.02	17.0815	0.383	0.7775	11.981	0.803	0.4615	0.4685	0.3885	0.4395	0.612	0.5615	0.8395	1.4805
TR01	Terrestrial Invertebrates	0.012	17.284	0.0355	0.6595	19.2585	0.08	0.0075	0.126	0.1015	0.0755	0.1235	0.1665	0.034	0.264
TR02	Terrestrial Invertebrates	0.0115	17.3915	0.0305	0.74	20.2165	0.0735	0.0055	0.087	0.108	0.0515	0.117	0.1575	0.033	0.278
TR03	Terrestrial Invertebrates	0.0115	15.59	0.0275	0.6365	18.6735	0.0775	0.007	0.176	0.1545	0.032	0.111	0.1545	0.035	0.2585
TR04	Terrestrial Invertebrates	0.012	16.968	0.02	0.736	20.21	0.077	0.007	0.144	0.108	0.06	0.127	0.189	0.066	0.27
TR05	Terrestrial Invertebrates	0.013	15.658	0.028	0.796	23.456	0.092	0.002	0.098	0.111	0.097	0.078	0.156	0.056	0.321
WBS-1L	Tree Swallow Liver	0.03	22.882	0.059	<MLOQ	4.068	0.012	0.009	0.003	0.119	0.012	0.113	0.488	0.037	0.156
WBS-3L	Tree Swallow Liver	0.048	28.128	0.048	0.206	5.949	0.021	0.086	<MLOQ	0.106	0.087	0.207	0.431	0.026	0.207
WBS-4L	Tree Swallow Liver	0.09	20.62	0.043	0.109	3.093	0.085	0.122	<MLOQ	0.129	<MLOQ	0.15	0.723	<MLOQ	0.15
WBS-7L	Tree Swallow Liver	0.004	26.702	0.033	0.168	4.433	0.076	0.015	<MLOQ	0.087	0.01	0.208	0.461	0.017	0.154

Sample ID	Compartment	iso16:0	16:0	16:1n11	16:1n9	16:1n7	7Me16:0	16:1n5	16:2n6	iso17:0	16:2n4	16:3n6	17:0	16:3n4	17:1
WBS-12L	Tree Swallow Liver	0.009	24.618	0.024	0.204	4.152	0.093	0.017	<MLOQ	0.141	0.051	0.144	0.484	0.027	0.17
WBS-13L	Tree Swallow Liver	0.005	23.394	0.046	0.141	3.047	0.078	0.022	<MLOQ	0.088	0.039	0.116	0.52	0.025	0.134
WBS-24L	Tree Swallow Liver	0.034	29.89	0.017	0.231	3.969	0.062	0.097	<MLOQ	0.1	<MLOQ	0.108	0.62	<MLOQ	0.154
WBS-29L	Tree Swallow Liver	0.004	29.36	0.017	0.205	6.405	0.101	0.11	0.009	0.017	0.004	0.253	0.48	0.009	0.227
WBS-32L	Tree Swallow Liver	0.036	20.791	0.049	0.191	3.172	0.073	0.084	<MLOQ	0.072	0.024	0.101	0.493	<MLOQ	0.104
WBS-37L	Tree Swallow Liver	0.007	27.567	0.015	0.191	6.04	0.095	0.019	<MLOQ	0.096	0.011	0.234	0.391	0.024	0.183
WBS-41L	Tree Swallow Liver	0.023	21.822	0.023	0.157	3.647	0.079	0.006	<MLOQ	0.081	0.022	0.148	0.519	0.021	0.128
WBS-49L	Tree Swallow Liver	0.008	22.083	0.031	0.203	3.976	0.109	0.011	<MLOQ	0.122	0.027	0.114	0.671	0.009	0.112
WBS-1C	Tree Swallow Carcass	0.05	20.418	0.1095	0.4885	18.388	0.2455	0.009	0.0375	0.0855	0.053	0.9245	0.392	0.2265	0.3885
WBS-3C	Tree Swallow Carcass	0.0585	21.2015	0.119	0.5655	17.6135	0.228	0.0165	0.03	0.084	0.0605	0.7815	0.4355	0.23	0.4395
WBS-4C	Tree Swallow Carcass	0.0585	22.4545	0.166	0.5105	15.945	0.2465	0.0655	0.0315	0.1295	0.0675	0.729	0.431	0.1745	0.5735
WBS-7C	Tree Swallow Carcass	0.0535	21.5	0.0995	0.525	19.175	0.2385	0.0125	0.034	0.081	0.05	0.897	0.352	0.233	0.338
WBS-12C	Tree Swallow Carcass	0.0605	22.4625	0.182	0.5755	17.3825	0.224	0.0075	0.0305	0.0845	0.0545	0.726	0.42	0.192	0.4335
WBS-13C	Tree Swallow Carcass	0.0625	22.739	0.1095	0.4295	18.066	0.276	0.074	0.0445	0.0945	0.059	1.023	0.4345	0.2365	0.41
WBS-18C	Tree Swallow Carcass	0.0555	22.046	0.0885	0.502	18.5025	0.2435	0.005	0.033	0.0935	0.0545	0.9255	0.398	0.229	0.359
WBS-24C	Tree Swallow Carcass	0.0565	20.891	0.112	0.491	16.587	0.233	0.0155	0.036	0.0865	0.0565	0.766	0.438	0.206	0.422
WBS-29C	Tree Swallow Carcass	0.047	21.9095	0.0755	0.553	18.8585	0.2585	0.0675	0.0425	0.094	0.059	0.9195	0.4315	0.2745	0.446
WBS-32C	Tree Swallow Carcass	0.047	22.2945	0.0855	0.5765	18.9035	0.24	0.0145	0.039	0.083	0.051	0.843	0.394	0.197	0.412
WBS-34C	Tree Swallow Carcass	0.0765	21.559	0.1515	0.4325	12.5095	0.2135	0.123	0.035	0.124	0.0585	0.6775	0.5265	0.1835	0.605
WBS-37C	Tree Swallow Carcass	0.0345	21.568	0.0785	0.4515	18.403	0.222	0.113	0.0405	0.0815	0.0555	0.9195	0.381	0.2535	0.3895
WBS-38C	Tree Swallow Carcass	0.031	23.191	0.089	0.4805	18.6065	0.251	0.13	0.04	0.107	0.065	0.953	0.427	0.238	0.5405
WBS-41C	Tree Swallow Carcass	0.0295	22.14	0.11	0.466	19.455	0.2605	0.0115	0.0395	0.091	0.057	0.989	0.4365	0.2525	0.513
WBS-49C	Tree Swallow Carcass	0.0475	22.0435	0.1075	0.579	17.617	0.2265	0.0145	0.0415	0.094	0.052	0.708	0.436	0.1445	0.468

Sample ID	Compartment	16:3n1	16:4n3	16:4n1	18:0	18:1n13	18:1n11	18:1n9	18:1n7	18:1n5	18:2D5,11	18:2n7	18:2n6	18:2n4	18:3n6
HD01	Aquatic Invertebrates	0.0065	0.0175	0.0225	5.223	0.27	0.1785	15.2305	7.7815	0.5505	0.281	0.1105	5.612	0.0795	0.1275
HD02	Aquatic Invertebrates	0.0095	0.021	0.025	4.777	0.245	0.148	14.5715	7.1135	0.4865	0.225	0.0935	6.38	0.0795	0.123
HD03	Aquatic Invertebrates	0.0065	0.028	0.031	4.672	0.2405	0.132	14.202	6.99	0.4805	0.232	0.094	6.539	0.083	0.1255
HD04	Aquatic Invertebrates	0.011	0.028	0.0275	4.82	0.2365	0.1755	14.517	6.9135	0.4685	0.2265	0.095	6.5525	0.0805	0.1195
HD05	Aquatic Invertebrates	0.0065	0.021	0.0255	4.6335	0.246	0.1405	14.813	7.032	0.4855	0.1265	0.095	6.585	0.079	0.1135
TR01	Terrestrial Invertebrates	0.002	0.0105	0.006	4.26	0.006	0.021	26.1595	0.766	0.0295	0.1845	0.0525	11.506	0.014	0.0355
TR02	Terrestrial Invertebrates	<MLOQ	0.0225	0.012	4.2975	0.01	0.035	25.04	0.759	0.0305	0.218	0.0695	11.3365	0.0115	0.0465
TR03	Terrestrial Invertebrates	<MLOQ	0.0125	0.009	4.999	0.006	0.0255	27.239	0.674	0.0275	0.295	0.0815	12.8725	0.0105	0.0515
TR04	Terrestrial Invertebrates	<MLOQ	0.011	0.012	4.505	0.017	0.045	24.955	0.968	0.039	0.019	0.072	11.896	0.01	0.078
TR05	Terrestrial Invertebrates	<MLOQ	0.024	0.008	4.386	0.012	0.033	25.424	0.949	0.032	0.188	0.078	12.802	0.006	0.032
WBS-1L	Tree Swallow Liver	<MLOQ	0.084	0.023	20.415	0.054	0.029	9.481	3.407	0.049	0.087	0.084	9.026	0.073	0.115
WBS-3L	Tree Swallow Liver	0.015	0.378	0.035	18.607	0.073	<MLOQ	11.368	3.59	0.065	0.138	0.11	7.122	0.089	0.133
WBS-4L	Tree Swallow Liver	0.06	0.042	0.007	20.35	<MLOQ	<MLOQ	7.507	3.148	0.057	0.224	0.18	8.841	0.105	0.161
WBS-7L	Tree Swallow Liver	0.008	0.046	0.021	20.538	0.048	0.023	8.026	3.618	0.053	0.127	0.093	8.075	0.106	0.1
WBS-12L	Tree Swallow Liver	0.01	0.047	<MLOQ	18.128	0.048	0.084	12.31	3.404	0.063	0.143	0.082	8.594	0.089	0.105
WBS-13L	Tree Swallow Liver	0.01	0.053	0.004	19.692	0.052	0.113	8.534	3.702	0.059	0.117	0.074	8.658	0.093	0.104
WBS-24L	Tree Swallow Liver	0.046	0.028	0.02	19.071	<MLOQ	0.108	10.451	3.238	0.044	0.227	0.133	7.637	0.092	0.157
WBS-29L	Tree Swallow Liver	0.007	0.029	0.006	16.559	0.033	0.023	11.474	3.93	0.064	0.052	0.097	8.127	0.104	0.075
WBS-32L	Tree Swallow Liver	0.007	0.073	0.01	19.153	0.072	0.072	8.183	3.275	0.059	0.094	0.074	7.817	0.075	0.08
WBS-37L	Tree Swallow Liver	0.008	0.03	0.008	17.729	0.053	0.05	13.753	3.774	0.068	0.078	0.089	6.984	0.104	0.076
WBS-41L	Tree Swallow Liver	MLOQ	0.042	<MLOQ	20.02	0.034	0.049	7.021	3.362	0.044	0.077	0.081	9.587	0.074	0.105
WBS-49L	Tree Swallow Liver	0.006	0.071	0.01	18.696	0.038	0.02	8.011	3.323	0.042	0.128	0.088	9.832	0.065	0.193
WBS-1C	Tree Swallow Carcass	0.0035	0.0235	0.0135	6.9575	0.0275	0.0405	14.5505	3.6145	0.0555	0.031	0.134	10.2185	0.0475	0.049
WBS-3C	Tree Swallow Carcass	0.0075	0.133	0.0065	8.4655	0.045	0.077	15.632	4.159	0.069	0.0355	0.132	10.14	0.0835	0.072
WBS-4C	Tree Swallow Carcass	0.006	0.182	0.006	8.2	0.04	0.0515	15.7675	3.512	0.067	0.0325	0.126	10.5765	0.0585	0.0805
WBS-7C	Tree Swallow Carcass	0.0055	0.129	0.006	7.4885	0.04	0.0595	17.0055	4.0805	0.0645	0.038	0.126	10.442	0.065	0.0545

Sample ID	Compartment	16:3n1	16:4n3	16:4n1	18:0	18:1n13	18:1n11	18:1n9	18:1n7	18:1n5	18:2D5,11	18:2n7	18:2n6	18:2n4	18:3n6
WBS-12C	Tree Swallow Carcass	0.0045	0.08	0.008	8.0815	0.048	0.0555	17.288	4.0015	0.0645	0.036	0.1255	10.705	0.0595	0.07
WBS-13C	Tree Swallow Carcass	0.003	0.377	0.0075	8.1805	0.036	0.034	13.5115	3.693	0.0685	0.0385	0.1295	10.289	0.062	0.061
WBS-18C	Tree Swallow Carcass	0.005	0.1635	0.0195	8.1655	0.0305	0.064	14.981	3.923	0.0735	0.041	0.135	10.353	0.0625	0.064
WBS-24C	Tree Swallow Carcass	0.005	0.3305	0.0095	8.07	0.0485	0.065	15.9255	4.1565	0.073	0.035	0.1285	10.697	0.06	0.079
WBS-29C	Tree Swallow Carcass	0.005	0.2785	0.0085	7.613	0.036	0.044	15.0905	3.929	0.0685	0.036	0.139	10.36	0.0575	0.0635
WBS-32C	Tree Swallow Carcass	0.005	0.3055	0.006	7.2855	0.0425	0.0395	16.7375	3.7685	0.0695	0.034	0.128	9.89	0.0525	0.0605
WBS-34C	Tree Swallow Carcass	0.0025	0.33	0.006	11.0385	0.0365	0.0665	14.156	4.675	0.0725	0.0445	0.131	11.2935	0.0705	0.087
WBS-37C	Tree Swallow Carcass	0.005	0.022	0.0075	8.2865	0.026	0.044	16.969	3.9495	0.062	0.0245	0.128	9.931	0.0635	0.083
WBS-38C	Tree Swallow Carcass	0.004	0.029	0.0085	8.202	0.0365	0.0525	14.5425	3.9665	0.067	0.032	0.1325	9.9615	0.0565	0.056
WBS-41C	Tree Swallow Carcass	0.006	0.0185	0.0085	7.797	0.035	0.0465	14.426	4.1975	0.062	0.0305	0.1355	10.646	0.059	0.075
WBS-49C	Tree Swallow Carcass	0.005	0.0145	0.007	7.689	0.037	0.0995	17.0305	3.5535	0.065	0.0425	0.1155	10.709	0.064	0.0785

Sample ID	Compartment	18:3n4	18:3n3	18:3n1	18:4n3	18:4n1	20:0	20:1n11	20:1n9	20:1n7	20:2n9	20:2n6	20:3n6	20:4n6	20:3n3
HD01	Aquatic Invertebrates	0.053	4.249	0.036	0.6235	0.1185	0.351	0.607	0.6675	0.131	0.031	0.629	0.297	3.496	0.2015
HD02	Aquatic Invertebrates	0.0425	5.82	0.039	0.777	0.1155	0.3715	0.52	0.6425	0.1355	0.001	0.5865	0.2765	3.6595	0.261
HD03	Aquatic Invertebrates	0.0565	5.646	0.0455	0.803	0.116	0.3735	0.6965	0.6305	0.1335	0.001	0.7725	0.3065	4.43	0.265
HD04	Aquatic Invertebrates	0.0605	5.8805	0.0555	0.8265	0.1125	0.415	0.5695	0.636	0.1145	<MLOQ	0.615	0.287	3.868	0.2595
HD05	Aquatic Invertebrates	0.049	6.2135	0.069	0.8505	0.1035	0.3765	0.5105	0.6275	0.115	<MLOQ	0.557	0.275	3.6845	0.272
TR01	Terrestrial Invertebrates	0.087	11.187	0.003	0.1555	0.055	0.435	0.0205	0.1685	0.134	0.1945	0.1945	0.056	0.5675	0.118
TR02	Terrestrial Invertebrates	0.061	11.658	<MLOQ	0.162	0.0605	0.41	0.026	0.1475	0.1875	0.187	0.187	0.0625	0.5695	0.0855
TR03	Terrestrial Invertebrates	0.153	9.512	<MLOQ	0.163	0.0515	0.4955	0.0175	0.1775	0.167	0.316	0.316	0.067	0.559	0.1045
TR04	Terrestrial Invertebrates	0.128	10.657	0.009	0.152	0.051	0.394	0.018	0.166	0.174	<MLOQ	0.164	0.052	0.605	0.106
TR05	Terrestrial Invertebrates	0.143	7.846	<MLOQ	0.132	0.061	0.354	0.019	0.129	0.228	<MLOQ	0.206	0.053	0.619	0.039
WBS-1L	Tree Swallow Liver	0.15	0.886	0.01	0.037	0.069	0.101	0.023	0.186	0.01	0.042	0.188	1.203	9.386	0.1
WBS-3L	Tree Swallow Liver	0.236	0.812	0.02	0.053	0.326	0.146	0.036	0.2	0.028	0.122	0.195	0.979	6.798	0.371
WBS-4L	Tree Swallow Liver	0.135	1.06	0.033	0.054	0.079	0.052	0.082	0.485	0.126	<MLOQ	0.248	1.316	9.383	0.144

Sample ID	Compartment	18:3n4	18:3n3	18:3n1	18:4n3	18:4n1	20:0	20:1n11	20:1n9	20:1n7	20:2n9	20:2n6	20:3n6	20:4n6	20:3n3
WBS-7L	Tree Swallow Liver	0.241	0.715	0.023	0.022	0.083	0.12	0.02	0.182	0.022	0.096	0.3	1.098	6.079	0.102
WBS-12L	Tree Swallow Liver	0.25	0.76	0.003	0.036	0.423	0.138	0.05	0.299	0.014	0.07	0.285	0.972	6.098	0.477
WBS-13L	Tree Swallow Liver	0.155	0.725	0.016	0.033	0.178	0.118	0.072	0.336	0.029	0.063	0.266	1.024	7.171	0.321
WBS-24L	Tree Swallow Liver	0.096	1.123	0.03	0.047	0.06	0.026	0.107	0.297	0.078	<MLOQ	0.217	0.786	5.5	0.084
WBS-29L	Tree Swallow Liver	0.204	1.184	0.012	0.031	0.113	0.098	0.014	0.157	0.022	0.044	0.169	0.629	4.55	0.108
WBS-32L	Tree Swallow Liver	0.155	0.69	0.01	0.078	0.635	0.124	0.105	0.342	0.04	0.021	0.203	1.135	8.674	0.807
WBS-37L	Tree Swallow Liver	0.222	0.881	0.015	0.044	0.221	0.146	0.025	0.222	0.034	0.089	0.219	0.755	4.628	0.146
WBS-41L	Tree Swallow Liver	0.136	1.033	0.023	0.046	0.285	0.088	0.009	0.139	0.02	0.022	0.219	1.276	9.806	0.228
WBS-49L	Tree Swallow Liver	0.139	1.062	0.007	0.035	0.122	0.16	0.019	0.193	0.017	0.089	0.216	1.66	10.386	0.193
WBS-1C	Tree Swallow Carcass	0.148	2.631	0.031	0.345	0.379	0.3225	0.031	0.24	0.029	0.013	0.0525	0.2255	1.1015	1.209
WBS-3C	Tree Swallow Carcass	0.163	2.7005	0.033	0.317	0.1885	0.448	0.048	0.303	0.0535	0.013	0.0665	0.369	2.133	0.1265
WBS-4C	Tree Swallow Carcass	0.1445	4.1395	0.021	0.319	0.0825	0.4615	0.0435	0.3085	0.0425	0.0135	0.056	0.283	1.593	0.0645
WBS-7C	Tree Swallow Carcass	0.1465	2.4845	0.0375	0.315	0.0885	0.376	0.04	0.3705	0.0465	0.0275	0.0555	0.254	1.4505	0.0345
WBS-12C	Tree Swallow Carcass	0.1435	3.0815	0.025	0.308	0.0945	0.3605	0.0435	0.3365	0.064	0.0275	0.0555	0.2635	1.5155	0.0395
WBS-13C	Tree Swallow Carcass	0.139	2.8185	0.025	0.315	0.0795	0.5075	0.043	0.2465	0.0495	0.0105	0.065	0.265	1.4745	0.0385
WBS-18C	Tree Swallow Carcass	0.1505	2.443	0.0425	0.3535	0.0765	0.448	0.065	0.342	0.0535	0.0405	0.052	0.3115	1.6615	0.0285
WBS-24C	Tree Swallow Carcass	0.1495	3.342	0.0335	0.3695	0.0785	0.441	0.0665	0.58	0.054	0.0255	0.074	0.2805	1.777	0.041
WBS-29C	Tree Swallow Carcass	0.158	2.878	0.037	0.3575	0.083	0.382	0.053	0.2795	0.0425	0.026	0.059	0.2495	1.5465	0.0365
WBS-32C	Tree Swallow Carcass	0.1335	2.839	0.03	0.3175	0.101	0.374	0.0505	0.403	0.047	0.0255	0.0545	0.269	1.5465	0.035
WBS-34C	Tree Swallow Carcass	0.1165	3.525	0.0215	0.2835	0.059	0.7295	0.0605	0.452	0.058	0.0495	0.0835	0.4515	2.8355	0.05
WBS-37C	Tree Swallow Carcass	0.1915	2.7535	0.0615	0.377	0.072	0.4145	0.0535	0.239	0.0475	0.03	0.057	0.258	1.389	0.0345
WBS-38C	Tree Swallow Carcass	0.134	3.3775	0.032	0.368	0.082	0.36	0.0385	0.308	0.0455	0.0205	0.0515	0.2495	1.5265	0.0385
WBS-41C	Tree Swallow Carcass	0.1535	3.055	0.04	0.388	0.0735	0.4105	0.042	0.2815	0.058	0.031	0.0595	0.24	1.4225	0.032
WBS-49C	Tree Swallow Carcass	0.1445	3.601	0.0215	0.2905	0.0835	0.5735	0.0445	0.3355	0.0475	0.0175	0.0595	0.3225	1.624	0.04

Sample ID	Compartment	20:4n3	20:5n3	22:1n11	22:1n9	22:1n7	22:2n6	21:5n3	22:4n6	22:5n6	22:4n3	22:5n3	22:6n3	24:1n9
HD01	Aquatic Invertebrates	0.191	6.674	0.057	0.1065	0.0285	0.0265	0.084	0.247	0.1725	0.0105	0.362	0.8925	0.009
HD02	Aquatic Invertebrates	0.195	7.483	0.0555	0.1305	0.02	0.019	0.1125	0.2285	0.1595	#DIV/0!	0.3745	1.0125	0.017
HD03	Aquatic Invertebrates	0.194	7.7825	0.2275	0.123	0.0285	0.0215	0.0835	0.2755	0.1805	0.0115	0.4245	0.9835	0.018
HD04	Aquatic Invertebrates	0.195	7.6315	0.0545	0.123	0.0235	0.013	0.0825	0.247	0.148	0.016	0.3815	1.0035	0.021
HD05	Aquatic Invertebrates	0.1985	7.565	0.0635	0.1235	0.024	0.0155	0.072	0.2235	0.1685	0.009	0.3625	0.9885	0.014
TR01	Terrestrial Invertebrates	0.045	1.448	0.0065	0.03	0.005	0.017	0.029	0.0215	0.0615	<MLOQ	<MLOQ	0.1005	<MLOQ
TR02	Terrestrial Invertebrates	0.0445	1.4785	0.0415	0.042	0.015	0.0345	0.0365	0.019	0.0155	<MLOQ	<MLOQ	0.078	<MLOQ
TR03	Terrestrial Invertebrates	0.0385	1.327	0.051	0.049	<MLOQ	0.0965	0.0595	0.017	0.0175	<MLOQ	<MLOQ	0.096	<MLOQ
TR04	Terrestrial Invertebrates	0.032	1.557	0.042	0.047	<MLOQ	0.022	0.051	0.021	0.021	<MLOQ	<MLOQ	0.086	<MLOQ
TR05	Terrestrial Invertebrates	0.024	1.225	0.025	0.021	<MLOQ	0.033	0.033	<MLOQ	0.015	<MLOQ	<MLOQ	0.083	<MLOQ
WBS-1L	Tree Swallow Liver	0.13	3.363	0.01	0.036	0.018	<MLOQ	0.022	0.47	0.107	0.012	3.91	7.303	0.014
WBS-3L	Tree Swallow Liver	0.037	0.021	0.04	0.028	0.07	0.023	0.025	0.536	0.128	0.032	3.95	5.451	0.019
WBS-4L	Tree Swallow Liver	0.144	4.246	0.866	<MLOQ	0.034	0.027	0.047	0.417	0.13	0.019	4.982	9.264	<MLOQ
WBS-7L	Tree Swallow Liver	0.145	3.266	0.014	0.035	0.046	0.025	0.031	0.446	0.08	0.028	5.451	6.322	0.016
WBS-12L	Tree Swallow Liver	0.17	3.677	0.072	0.063	0.044	<MLOQ	0.016	0.398	0.07	0.015	4.457	6.173	0.017
WBS-13L	Tree Swallow Liver	0.13	4.191	0.184	0.084	0.043	<MLOQ	0.03	0.493	0.132	0.019	5.393	8.603	0.015
WBS-24L	Tree Swallow Liver	0.112	2.037	0.317	<MLOQ	0.029	0.019	0.054	0.458	0.144	0.022	4.605	6.284	0.094
WBS-29L	Tree Swallow Liver	0.108	2.266	0.003	0.018	0.027	0.006	0.02	0.362	0.067	0.021	5.029	4.735	0.017
WBS-32L	Tree Swallow Liver	0.192	8.314	0.232	0.11	0.022	0.014	0.006	0.452	0.081	<MLOQ	4.289	8.081	<MLOQ
WBS-37L	Tree Swallow Liver	0.159	2.902	<MLOQ	0.033	0.047	<MLOQ	0.017	0.377	0.078	0.025	4.077	4.882	0.011
WBS-41L	Tree Swallow Liver	0.145	4.764	0.004	0.037	0.025	<MLOQ	0.027	0.466	0.095	0.01	4.549	8.433	0.011
WBS-49L	Tree Swallow Liver	0.076	4	<MLOQ	0.085	0.049	0.121	0.012	0.499	0.125	0.017	4.642	6.937	0.03
WBS-1C	Tree Swallow Carcass	0.15	7.685	0.004	0.027	0.008	0.0635	0.112	0.106	0.007	0.011	0.9545	0.597	<MLOQ
WBS-3C	Tree Swallow Carcass	0.0785	2.8095	0.004	0.0455	0.018	0.04	0.235	0.2855	0.0245	<MLOQ	1.4095	1.007	<MLOQ
WBS-4C	Tree Swallow Carcass	0.0935	2.758	0.004	0.0385	0.0075	0.011	0.14	0.1545	0.011	<MLOQ	1.1675	0.8395	<MLOQ
WBS-7C	Tree Swallow Carcass	0.083	2.4895	<MLOQ	0.051	0.011	0.026	0.113	0.158	0.0125	<MLOQ	1.2585	0.7865	<MLOQ

Sample ID	Compartment	20:4n3	20:5n3	22:1n11	22:1n9	22:1n7	22:2n6	21:5n3	22:4n6	22:5n6	22:4n3	22:5n3	22:6n3	24:1n9
WBS-12C	Tree Swallow Carcass	0.0815	2.005	<MLOQ	0.0445	0.009	0.0165	0.1515	0.1875	0.008	<MLOQ	1.111	0.747	<MLOQ
WBS-13C	Tree Swallow Carcass	0.1345	3.262	0.067	0.0385	0.0115	#DIV/0!	0.156	0.1535	0.007	<MLOQ	1.425	0.917	<MLOQ
WBS-18C	Tree Swallow Carcass	0.135	3.0155	0.172	0.047	0.008	0.012	0.0895	0.1785	0.012	<MLOQ	1.324	1.0365	<MLOQ
WBS-24C	Tree Swallow Carcass	0.1375	3.2175	0.191	0.0695	0.0135	0.0175	0.05	0.202	0.0245	<MLOQ	1.496	1.0855	<MLOQ
WBS-29C	Tree Swallow Carcass	0.133	2.827	0.0785	0.036	0.011	0.0095	0.061	0.172	0.019	<MLOQ	1.378	0.9025	<MLOQ
WBS-32C	Tree Swallow Carcass	0.1245	2.4215	0.1	0.0505	0.0095	0.019	0.0685	0.1685	0.018	<MLOQ	1.324	0.929	<MLOQ
WBS-34C	Tree Swallow Carcass	0.154	2.6145	0.0305	0.0595	0.0155	0.02	0.095	0.3535	0.0385	0.049	1.8015	1.2095	<MLOQ
WBS-37C	Tree Swallow Carcass	0.129	2.275	0.011	0.028	0.012	0.017	0.199	0.192	0.007	<MLOQ	1.034	0.8305	<MLOQ
WBS-38C	Tree Swallow Carcass	0.1375	2.5795	0.004	0.0365	0.0095	0.018	0.1905	0.1635	0.021	<MLOQ	1.256	0.7945	<MLOQ
WBS-41C	Tree Swallow Carcass	0.1295	2.659	0.014	0.034	0.0115	<MLOQ	0.174	0.161	0.01	<MLOQ	1.1205	0.701	<MLOQ
WBS-49C	Tree Swallow Carcass	0.1655	2.271	<MLOQ	0.044	0.015	<MLOQ	0.1485	0.1605	0.0115	<MLOQ	1.129	0.786	<MLOQ

Table S12 - Concentrations (ng/g in air, ng/mL in surface water and rinsate, ng/g dw in sediment, and ng/g ww in biota samples) of 46 per- and polyfluoroalkyl substances identified in passive air, surface water, sediment, aquatic invertebrates, terrestrial invertebrates, and tree swallow GI-Tracts and livers collected from an aquatic-terrestrial food web.

Data on PFAS concentrations in the gastrointestinal tract contents generated during this study are available as a USGS data release[9].

Sample ID	Compartment	PFBA	PFPeA	PFHxA	PFHpA	PFOA	PFNA	PFDA	PFUnA	PFDoA	PFTrDA	PFTeDA
WBS-1-1	Tree Swallow Liver	ND	ND	ND	ND	ND	1.54	2.63	0.93	ND	ND	ND
WBS-3-1	Tree Swallow Liver	ND	ND	ND	ND	ND	0.98	1.78	0.86	ND	ND	ND
WBS-4-1	Tree Swallow Liver	ND	ND	ND	ND	ND	1.34	2.62	0.725	ND	ND	ND
WBS-7-1	Tree Swallow Liver	ND	ND	ND	ND	ND	1.37	2.44	0.871	ND	ND	ND
WBS-12-1	Tree Swallow Liver	ND	ND	ND	ND	ND	1.06	2.3	0.822	ND	ND	ND
WBS-13-1	Tree Swallow Liver	ND	ND	ND	ND	ND	2.08	4.09	1.23	ND	ND	ND
WBS-18-1	Tree Swallow Liver	ND	ND	ND	ND	ND	1.43	3.1	0.85	ND	ND	ND
WBS-24-1	Tree Swallow Liver	2.3	ND	ND	ND	ND	1.12	2.9	0.884	ND	ND	ND
WBS-29-1	Tree Swallow Liver	2.31	ND	ND	ND	ND	1.49	3.44	0.998	ND	ND	ND
WBS-32-1	Tree Swallow Liver	ND	ND	ND	ND	ND	1.41	2.97	1.01	ND	ND	ND
WBS-34-1	Tree Swallow Liver	2.26	ND	ND	ND	ND	1.04	2.09	0.75	ND	ND	ND
WBS-37-1	Tree Swallow Liver	2.3	ND	ND	ND	ND	1.02	1.99	0.715	ND	ND	ND
WBS-38-1	Tree Swallow Liver	ND	ND	ND	ND	ND	1.28	3.05	0.847	ND	ND	ND
WBS-41-1	Tree Swallow Liver	ND	ND	ND	ND	ND	1.19	2.64	0.817	0.524	0.51	ND
WBS-49-1	Tree Swallow Liver	ND	ND	ND	ND	ND	1.33	2.45	0.783	ND	ND	ND
WBS-1-1	Tree Swallow GI-Tract	ND	ND	ND	ND	ND	0.39	0.971	0.367	0.168	0.146	0.165
WBS-3-1	Tree Swallow GI-Tract	ND	ND	ND	ND	0.153	0.259	0.441	0.245	0.143	0.152	0.126
WBS-4-1	Tree Swallow GI-Tract	ND	0.209	ND	ND	ND	0.366	0.952	0.329	0.163	0.145	0.153
WBS-7-1	Tree Swallow GI-Tract	ND	ND	ND	ND	0.11	0.422	0.858	0.393	0.25	0.174	0.208
WBS-12-1	Tree Swallow GI-Tract	ND	ND	ND	ND	0.122	0.269	0.711	0.281	0.14	0.156	0.162
WBS-13-1	Tree Swallow GI-Tract	ND	ND	ND	ND	0.13	0.609	1.35	0.49	0.204	0.176	0.163

Sample ID	Compartment	PFBA	PFPeA	PFHxA	PFHpA	PFOA	PFNA	PFDA	PFUnA	PFDoA	PFTrDA	PFTeDA
WBS-18-1	Tree Swallow GI-Tract	ND	ND	ND	ND	ND	0.356	0.847	0.381	0.195	0.148	0.147
WBS-24-1	Tree Swallow GI-Tract	ND	ND	ND	ND	0.103	0.284	0.646	0.284	0.179	0.168	0.145
WBS-32-1	Tree Swallow GI-Tract	ND	ND	ND	ND	0.117	0.42	0.956	0.435	0.176	0.185	0.21
WBS-34-1	Tree Swallow GI-Tract	ND	ND	ND	ND	ND	0.12	0.502	0.261	0.17	0.177	0.143
WBS-37-1	Tree Swallow GI-Tract	ND	ND	ND	ND	ND	0.308	0.643	0.34	0.234	0.204	0.236
WBS-38-1	Tree Swallow GI-Tract	ND	ND	ND	ND	ND	0.371	0.988	0.368	0.179	0.142	0.137
WBS-41-1	Tree Swallow GI-Tract	ND	ND	ND	ND	ND	0.363	0.864	0.387	0.367	0.239	0.319
WBS-49-1	Tree Swallow GI-Tract	ND	ND	ND	ND	ND	0.338	0.729	0.315	0.16	0.147	0.17
WBS-S01	Sediment	ND	ND	0.656	ND	0.378	ND	0.529	ND	0.902	0.383	0.436
WBS-S02	Sediment	ND	ND	0.951	ND	0.135	ND	0.316	ND	0.408	0.288	0.316
WBS-S03	Sediment	ND	ND	0.357	ND	0.134	ND	0.209	0.139	0.313	0.144	0.252
WBS-S04	Sediment	ND	ND	0.223	ND	0.105	ND	0.125	0.086	0.211	0.102	0.134
WBS-S05	Sediment	ND	ND	1.59	ND	0.529	ND	0.764	ND	1.02	0.49	0.775
WBS-S06	Sediment	ND	ND	1.71	ND	0.19	ND	0.706	ND	1.02	0.23	0.905
WBS-W01	Water	0.0126	0.0139	0.0163	0.00374	0.00621	0.000976	0.00132	ND	ND	ND	ND
WBS-W02	Water	0.0118	0.0136	0.016	0.00417	0.00638	0.00114	0.00101	ND	ND	ND	ND
WBS-W03	Water	0.012	0.014	0.0177	0.00379	0.00571	0.000987	0.00142	ND	ND	ND	ND
WBS-W04	Water	0.0123	0.0147	0.0194	0.00332	0.00519	0.00106	0.00116	ND	ND	ND	ND
WBS-W05	Water	0.0118	0.0145	0.0178	0.00413	0.00605	0.001	0.000933	ND	ND	ND	ND
WBS-W06	Water	0.0123	0.0139	0.0181	0.00332	0.00632	0.00112	0.00164	ND	ND	ND	ND
WBS-R01	Rinsate Water	0.0193	0.0163	0.019	0.00431	0.00778	0.00198	0.00219	ND	0.000515	ND	ND
WBS-R02	Rinsate Water	0.0135	0.0145	0.0163	0.00469	0.0067	0.0015	0.00177	ND	0.00064	ND	0.000498
WBS-T01	Terrestrial Invertebrates	ND	ND	ND	ND	ND	ND	ND	ND	ND	ND	ND
WBS-T02	Terrestrial Invertebrates	ND	ND	ND	ND	ND	ND	ND	ND	ND	ND	ND
WBS-T03	Terrestrial Invertebrates	ND	ND	ND	ND	ND	ND	ND	ND	ND	ND	ND
WBS-T04	Terrestrial Invertebrates	ND	ND	ND	ND	ND	ND	ND	ND	ND	ND	ND

Sample ID	Compartment	PFBA	PFPeA	PFHxA	PFHpA	PFOA	PFNA	PFDA	PFUnA	PFDoA	PFTrDA	PFTeDA
WBS-T05	Terrestrial Invertebrates	ND	ND	ND	ND	ND	ND	ND	ND	ND	ND	ND
WBS-HD01	Aquatic Invertebrates	ND	0.194	0.271	ND	0.101	ND	ND	ND	0.374	0.248	0.423
WBS-HD02	Aquatic Invertebrates	ND	ND	0.221	ND	0.114	ND	0.1	ND	0.44	0.259	0.25
WBS-HD03	Aquatic Invertebrates	ND	0.202	0.157	ND	0.088	ND	ND	ND	0.37	0.22	0.201
WBS-HD04	Aquatic Invertebrates	ND	0.218	0.157	ND	0.1	ND	0.101	ND	0.455	0.251	0.242
WBS-HD05	Aquatic Invertebrates	ND	ND	0.361	ND	0.0855	ND	0.101	ND	0.35	0.222	0.237
WBS-SIP1	Air	0.04095966	ND	0.03092762	1.810E-06	0.00595972	0.0011394	0.00181754	ND	ND	0.00213614	ND
WBS-SIP2	Air	0.04610344	ND	0.01833761	0.00109478	0.00529753	8.486E-06	0.00242338	ND	ND	0.00160211	ND
WBS-SIP3	Air	0.04210273	ND	0.006295	0.00218957	0.00463534	0.0011394	0.00181754	ND	ND	0.00213614	ND
WBS-SIP4	Air	0.08439596	ND	0.08074024	0.00437913	0.00993287	0.0017091	0.00363508	ND	ND	0.00160211	ND
WBS-SIP5	Air	0.06496393	ND	0.01176892	0.00164217	0.00860849	0.0011394	0.00242338	ND	ND	0.00160211	ND

Sample ID	Compartment	PFBS	PFPeS	PFHxS	PFHpS	PFOS	PFNS	PFDS	4:2 FTS	6:2 FTS	8:2 FTS	10:2 FTS
WBS-1-1	Tree Swallow Liver	ND	ND	ND	ND	28.90	ND	ND	ND	ND	ND	NM
WBS-3-1	Tree Swallow Liver	ND	ND	ND	ND	26.50	ND	ND	ND	ND	ND	NM
WBS-4-1	Tree Swallow Liver	ND	ND	ND	ND	29.20	ND	ND	ND	ND	ND	NM
WBS-7-1	Tree Swallow Liver	ND	ND	ND	ND	23.40	ND	ND	ND	ND	ND	NM
WBS-12-1	Tree Swallow Liver	ND	ND	ND	ND	23.90	ND	ND	ND	ND	ND	NM
WBS-13-1	Tree Swallow Liver	ND	ND	ND	ND	49.10	ND	ND	ND	ND	ND	NM
WBS-18-1	Tree Swallow Liver	ND	ND	ND	ND	30.10	ND	ND	ND	ND	ND	NM
WBS-24-1	Tree Swallow Liver	ND	ND	ND	ND	29.30	ND	ND	ND	ND	ND	NM
WBS-29-1	Tree Swallow Liver	ND	ND	ND	ND	32.70	ND	ND	ND	ND	ND	NM
WBS-32-1	Tree Swallow Liver	ND	ND	ND	ND	32.90	ND	ND	ND	ND	ND	NM
WBS-34-1	Tree Swallow Liver	ND	ND	ND	ND	21.80	ND	0.62	ND	ND	ND	NM
WBS-37-1	Tree Swallow Liver	ND	ND	ND	ND	23.00	ND	ND	ND	ND	ND	NM
WBS-38-1	Tree Swallow Liver	ND	ND	ND	ND	29.20	ND	ND	ND	ND	ND	NM

Sample ID	Compartment	PFBS	PFPeS	PFHxS	PFHpS	PFOS	PFNS	PFDS	4:2 FTS	6:2 FTS	8:2 FTS	10:2 FTS
WBS-41-1	Tree Swallow Liver	ND	ND	ND	ND	27.10	ND	ND	ND	ND	ND	NM
WBS-49-1	Tree Swallow Liver	ND	ND	ND	ND	30.60	ND	ND	ND	ND	ND	NM
WBS-1-1	Tree Swallow GI-Tract	ND	ND	ND	ND	10.60	ND	0.16	ND	ND	ND	NM
WBS-3-1	Tree Swallow GI-Tract	ND	ND	ND	ND	6.00	ND	0.12	ND	ND	ND	NM
WBS-4-1	Tree Swallow GI-Tract	ND	ND	ND	ND	10.30	ND	0.17	ND	ND	ND	NM
WBS-7-1	Tree Swallow GI-Tract	ND	ND	ND	ND	9.55	ND	0.13	ND	ND	ND	NM
WBS-12-1	Tree Swallow GI-Tract	ND	ND	ND	ND	8.03	ND	0.16	ND	ND	ND	NM
WBS-13-1	Tree Swallow GI-Tract	ND	ND	ND	ND	14.90	ND	0.14	ND	ND	ND	NM
WBS-18-1	Tree Swallow GI-Tract	ND	ND	ND	ND	10.20	ND	0.20	ND	ND	ND	NM
WBS-24-1	Tree Swallow GI-Tract	ND	ND	ND	0.12	8.15	ND	0.19	ND	ND	ND	NM
WBS-32-1	Tree Swallow GI-Tract	ND	ND	ND	ND	11.70	ND	0.16	ND	ND	ND	NM
WBS-34-1	Tree Swallow GI-Tract	ND	ND	ND	ND	6.07	ND	0.21	ND	ND	ND	NM
WBS-37-1	Tree Swallow GI-Tract	ND	ND	ND	ND	8.51	ND	0.19	ND	ND	ND	NM
WBS-38-1	Tree Swallow GI-Tract	ND	ND	ND	ND	11.80	ND	0.22	ND	ND	ND	NM
WBS-41-1	Tree Swallow GI-Tract	ND	ND	ND	ND	9.85	ND	0.17	ND	ND	ND	NM
WBS-49-1	Tree Swallow GI-Tract	ND	ND	ND	0.13	9.20	ND	0.14	ND	ND	ND	NM
WBS-S01	Aquatic Sediment	ND	ND	ND	ND	1.74	ND	0.42	ND	ND	ND	NM
WBS-S02	Aquatic Sediment	ND	ND	ND	ND	1.43	ND	0.25	ND	ND	ND	NM
WBS-S03	Aquatic Sediment	ND	ND	ND	ND	1.14	0.13	0.56	ND	ND	ND	NM
WBS-S04	Aquatic Sediment	ND	ND	ND	ND	0.51	0.08	0.28	ND	ND	ND	NM
WBS-S05	Aquatic Sediment	ND	ND	ND	ND	2.70	ND	0.76	ND	ND	ND	NM
WBS-S06	Aquatic Sediment	ND	ND	ND	ND	4.4	ND	0.78	ND	ND	ND	NM
WBS-W01	Water	0.00447	0.00047	0.00202	ND	0.0059	ND	ND	ND	0.00607	ND	NM
WBS-W02	Water	0.00374	0.000449	0.00209	ND	0.00599	ND	ND	ND	0.00696	ND	NM
WBS-W03	Water	0.00403	0.00	0.00236	ND	0.00621	ND	ND	ND	0.00574	ND	NM
WBS-W04	Water	0.00381	ND	0.00211	ND	0.00566	ND	ND	ND	0.00563	ND	NM

Sample ID	Compartment	PFBS	PFPeS	PFHxS	PFHpS	PFOS	PFNS	PFDS	4:2 FTS	6:2 FTS	8:2 FTS	10:2 FTS
WBS-W05	Water	0.00348	0.00	0.0023	ND	0.0054	ND	ND	ND	0.00609	ND	NM
WBS-W06	Water	0.00406	ND	0.00207	ND	0.0067	ND	ND	ND	0.00576	ND	NM
WBS-R01	Water	0.00445	ND	0.00229	ND	0.34	0.00	ND	ND	0.00857	ND	NM
WBS-R02	Water	0.00	ND	0.00	ND	0.03	ND	ND	ND	0.01	ND	NM
WBS-T01	Terrestrial Invertebrates	ND	ND	ND	ND	1.90	ND	ND	ND	0.387	ND	NM
WBS-T02	Terrestrial Invertebrates	ND	ND	ND	ND	1.91	ND	ND	ND	0.32	ND	NM
WBS-T03	Terrestrial Invertebrates	ND	ND	ND	ND	1.79	ND	ND	ND	0.36	ND	NM
WBS-T04	Terrestrial Invertebrates	ND	ND	ND	ND	1.97	ND	ND	ND	0.36	ND	NM
WBS-T05	Terrestrial Invertebrates	ND	ND	ND	ND	2.83	ND	ND	ND	0.52	ND	NM
WBS-HD01	Aquatic Invertebrates	ND	ND	ND	ND	1.28	ND	0.12	ND	ND	ND	NM
WBS-HD02	Aquatic Invertebrates	ND	ND	ND	ND	1.54	ND	0.12	ND	ND	ND	NM
WBS-HD03	Aquatic Invertebrates	ND	ND	ND	ND	1.32	ND	0.13	ND	ND	ND	NM
WBS-HD04	Aquatic Invertebrates	ND	ND	ND	ND	1.57	ND	0.11	ND	4.86	ND	NM
WBS-HD05	Aquatic Invertebrates	ND	ND	ND	ND	1.6000	ND	0.1070	ND	ND	ND	NM
WBS-SIP1	Air	0.0040	NM	0.0007	NM	0.0558	NM	ND	NM	0.0000	0.0002	0.0000
WBS-SIP2	Air	0.0040	NM	0.0007	NM	0.0467	NM	ND	NM	0.0046	0.0003	0.0002
WBS-SIP3	Air	0.0047	NM	0.0014	NM	0.0387	NM	ND	NM	0.0025	0.0000	0.0000
WBS-SIP4	Air	0.0047	NM	0.0029	NM	0.2564	NM	ND	NM	0.0032	0.0000	0.0009
WBS-SIP5	Air	0.00201364	NM	0	NM	0.08692	NM	ND	NM	0.00309	2.0367E-05	5.9921E-05

References

1. Kielstra, B. W., Arnott, S. E., & Gunn, J. M. (2017). Subcatchment deltas and upland features influence multiscale aquatic ecosystem recovery in damaged landscapes. *Ecological Applications*, *27*(8), 2249-2261.

2. Graves, S. D., Liber, K., Palace, V., Hecker, M., Doig, L. E., & Janz, D. M. (2019). Effects of selenium on benthic macroinvertebrates and fathead minnow (*Pimephales promelas*) in a boreal lake ecosystem. *Ecotoxicology and Environmental Safety*, *182*, 109354.

3. Custer, C. M., Custer, T. W., Dummer, P. M., Schultz, S., Tseng, C. Y., Karouna-Renier, N., & Matson, C. W. (2020). Legacy and contaminants of emerging concern in tree swallows along an agricultural to industrial gradient: Maumee River, Ohio. *Environmental Toxicology and Chemistry*, *39*(10), 1936-1952.

4. Sun, J., Letcher, R. J., Eens, M., Covaci, A., & Fernie, K. J. (2020). Perfluoroalkyl acids and sulfonamides and dietary, biological and ecological associations in peregrine falcons from the Laurentian Great Lakes Basin, Canada. *Environmental Research*, *191*, 110151.

5. Sherlock, C., Fernie, K. J., Munno, K., Provencher, J., & Rochman, C. (2022). The potential of aerial insectivores for monitoring microplastics in terrestrial environments. *Science of The Total Environment*, *807*, 150453.

6. Hopkins, K. E., McKinney, M. A., Letcher, R. J., & Fernie, K. J. (2023). The influence of environmental and ecological factors on the accumulation and distribution of short-and long-chain perfluoroalkyl acids in a mid-trophic avian insectivore. *Environmental Pollution*, 121133.

7. Pedro, S., Fisk, A. T., Ferguson, S. H., Hussey, N. E., Kessel, S. T., & McKinney, M. A. (2020). Broad feeding niches of capelin and sand lance may overlap those of polar cod and other native fish in the eastern Canadian Arctic. *Polar Biology*, *43*, 1707-1724.

8. Rauert, C., Shoieb, M., Schuster, J. K., Eng, A., & Harner, T. (2018). Atmospheric concentrations and trends of poly-and perfluoroalkyl substances (PFAS) and volatile methyl siloxanes (VMS) over 7 years of sampling in the Global Atmospheric Passive Sampling (GAPS) network. *Environmental Pollution*,*238*, 94-102.

9.Karouna-Renier, N.K., Hopkins, K.E., Fernie, K.J. (2023). Per- and polyfluoroalkyl Substances in tree swallow gut contents. USGS Data Release doi :5066/P92J8EQ
